# Allatotropin: A pleiotropic neuropeptide that elicits mosquito immune responses

**DOI:** 10.1371/journal.pone.0175759

**Published:** 2017-04-20

**Authors:** Salvador Hernández-Martínez, Minerva Sánchez-Zavaleta, Kevin Brito, Antonia Herrera-Ortiz, Sheila Ons, Fernando G. Noriega

**Affiliations:** 1 Centro de Investigaciones Sobre Enfermedades Infecciosas, Instituto Nacional de Salud Pública, Cuernavaca, Morelos, México; 2 Laboratorio de Neurobiología de Insectos. Centro Regional de Estudios Genómicos. Universidad Nacional de La Plata. La Plata, Argentina; 3 Department of Biological Sciences and Biomolecular Science Institute, Florida International University. Miami, FL, United States of America; Kansas State University, UNITED STATES

## Abstract

Allatotropins (AT) are neuropeptides with pleotropic functions on a variety of insect tissues. They affect processes such as juvenile hormone biosynthesis, cardiac rhythm, oviduct and hindgut contractions, nutrient absorption and circadian cycle. The present work provides experimental evidence that AT elicits immune responses in two important mosquito disease vectors, *Anopheles albimanus* and *Aedes aegypti*. Hemocytes and an immune-competent mosquito cell line responded to AT by showing strong morphological changes and increasing bacterial phagocytic activity. Phenoloxidase activity in hemolymph was also increased in *Ae*. *aegypti* mosquitoes treated with AT but not in *An*. *albimanus*, suggesting differences in the AT-dependent immune activation in the two species. In addition, two important insect immune markers, nitric oxide levels and expression of antimicrobial peptide genes, were increased in *An*. *albimanus* guts after AT treatment. AT conjugated to quantum dot nanocrystals (QDots) specifically labeled hemocytes *in vivo* in both mosquito species, implying molecular interactions between AT and hemocytes. The results of our studies suggest a new role for AT in the modulation of the immune response in mosquitoes.

## Introduction

Insect neuropeptides act as neuromodulators in the central and peripheral nervous system, as well as regulatory hormones released into the circulation. The functional roles of insect neuropeptides include regulation of homeostasis, organization of behaviors, initiation and modulation of neuronal and muscular activity and coordination of developmental processes and reproduction [1]. In the malaria mosquito *Anopheles gambiae*, at least 35 genes encoding peptide precursors and 40 putative peptide G-protein-coupled receptors (GPCRs) were identified [2]. In the *Aedes aegypti* genome 43 neuropeptides [3], and over 40 GPCRs [4] have been described.

Allatotropins (AT) are peptides originally identified by their stimulatory effect on juvenile hormone (JH) biosynthesis [5]; in addition, they affect a variety of processes in different tissues, including modulation of cardiac rhythm, oviduct and hindgut contractions, nutrient absorption and circadian cycle [6]. *Ae*. *aegypti* allatotropin (*Aedes*-AT) was isolated from abdomens and thoraces of mosquitoes, and its structure determined to be Ala-Pro-Phe-Arg-Asn-Ser-Glu-Met-Met-Thr-Ala-Arg-Gly-Phe-amide [7]; this amino acid (AA) sequence is identical to the allatotropin AA sequences of *An*. *gambiae* (AGAP012130-PA) and *Anopheles albimanus* (AALB004051-PA). *Aedes*-AT immunoreactivity was detected in cells in brain and ventral ganglia, as well as in nerve projections innervating different thoracic and abdominal tissues of *Ae*. *aegypti* and *An*. *albimanus*, including release-like sites in the heart and gut [8]. *Aedes*-AT stimulates JH biosynthesis by the mosquito CA [9, 10]; the *Aedes*-AT receptor (AeATr) was identified and functional characterized; analysis of the tissue distribution of AeATr mRNA in adult female *Ae*. *aegypti* revealed high transcript levels in the nervous system (brain, abdominal, thoracic and ventral ganglia), *corpora allata*-*corpora cardiaca* complex and ovary. The receptor is also expressed in heart and hindgut, as well as testis and accessory glands of male mosquitoes [11]. The *An*. *albimanus* AT receptor AA sequence (AALB007506-PA) shares 90.2% identity with the AeATr sequence, suggesting an evolutionary conservation of AT signaling in both mosquito species.

The mosquito innate immune system is composed of both cellular and humoral factors, where the participation of hemolymphatic cells called hemocytes is essential [12]. Humoral defenses include the production of soluble effector molecules such as antimicrobial peptides, reactive oxygen species and enzymatic cascades that regulate coagulation and melanization in the hemolymph [13]. Cellular defenses refer to immune responses, including pathogen recognition, phagocytosis and encapsulation that are mediated by hemocytes [14]. The regulation of immune responses is a critical process that is fine-tuned to prevent harmful deficiencies or excesses in the responses. Immune response regulation (amplification or suppression) is often based in signal modulation. The recognition of PAMPs (Pathogen Associated Molecular Patterns) by PRRs (Patterns Recognition Receptors), either present in the hemolymph or anchored on hemocyte membranes, triggers the activation of serine protease cascades that amplify danger signals and activate effector mechanisms [12]. The participation of neuroendocrine factors regulating immune responses in insects have been previously reported in cockroaches and locusts [15, 16, 17], although the biological relevance and mechanisms involved on this neuroendocrine control of the immune responses remain unclear. The present work provides experimental evidence that AT elicits immune responses in hemocytes and guts of mosquitoes.

## Materials and methods

### Mosquitoes

White-striped pupal-phenotype *An*. *albimanus* and *Ae*. *aegypti* (Rockefeller strain) were raised at 28°C, 80% relative humidity, with a 12-h light/12-h dark photoperiod. Adult females were fed ad libitum with cotton pads soaked with 3% sucrose solution [18]. In this study only 2- and 3-day-old adult females of *An*. *albimanus* and *Ae*. *aegypti* were used.

### Peptides

Synthetic *Aedes*-AT (APFRNSEMMTARGF) [7] was custom-synthesized by Alpha Diagnostic International (San Antonio, TX), purified by reversed phase liquid chromatography, and assessed to be 97% pure by analytical reversed phase liquid chromatography and MALDI-TOF MS. *Ae*. *aegypti* NPLP1 (neuropeptide like precursor 1; SYRSLLRDGATF) [19] was synthesized by Peptide 2.0 (Chantilly, VA) with a purity of 81%. Stock aqueous solutions of synthetic peptides (10^−4^ M) were stored in aliquots at -80°C. For each assay, a new stock was utilized.

### Peptide conjugation to quantum dot nanocrystals (QD)

Streptavidin-conjugated QD (1 μM; emissions at 605 nm) were purchased from Quantum Dot Co. (Hayward, CA). Each QD contains 5–10 streptavidin molecules, with a total of 20–40 binding sites for biotin. A biotinylated AT peptide was custom-synthesized by Biopeptide Co. (San Diego, CA). Biotinylated peptide stocks were prepared at 0.92 mM in dimethylsulfoxide (DMSO, Sigma, St. Louis, MO), and aliquots stored at -80°C. AT conjugation to QD was performed as previously reported [20]. Briefly, an excess of biotinylated peptide (4.3 μl from the 0.92 mM stock) were added to 10 μl of streptavidin-conjugated QD (1 μM), along with 58.7 μl of incubation buffer (2% bovine serum albumin in 50 mM borate buffer pH 8.3, containing 0.05% sodium azide). The mixture was incubated for 1.5 h at room temperature (RT). The peptide excess was removed by centrifugation in a Microcon-100 concentrator (Amicon Bio separations, Bedford, MA) at 16,000 *g* with 0.5 ml of phosphate-buffered saline (PBS) (140 mM NaCl, 2.6 mM KCl, 1.5 mM KH_2_PO4, 20.4 mM Na_2_HPO_4_, pH 7.2), followed by 5 washes with PBS (5 min each). The AT-QD (now referred as “AT-QD conjugates”) were recovered from the top of the Microcon membrane in 100 μl of PBS and stored at 4°C. The AT-QD conjugates stock concentration was 0.1 μM. The average diameter of QD-AT conjugates was 30–32 nm.

### Mosquito inoculations

Mosquito inoculations were carried out as previously described [21]. Briefly, fine needles were made from 100 μl micro-glass capillary tubes using a pipette puller P-30 (Sutter Instrument, Novato, CA), and mounted on a pipette pump (Drummond, Broomall, PA). Mosquitoes were cold-anesthetized on ice and their abdomens washed with 70% ethanol and air dried. Inoculations of 0.25 μl of specific solutions were carried out through the pleural membrane, between the fourth and fifth abdominal segments. Mosquitoes were allowed to recover before dissecting or perfusing them.

### Hemocyte collection

Hemocytes were obtained by perfusion as previously described [22]. Needles similar to those used for inoculations were utilized for perfusions. Mosquito abdomens were washed with 70% ethanol and air dried. A small tear was made laterally on the intersegmentary membrane of the last abdominal segment. Needles were inserted manually through the neck membrane into the thoracic cavity, and mosquitoes were perfused with freshly prepared bleeding solution [Grace’s insect medium with L-glutamine (G-9771, Sigma, St. Louis, MO) plus 0.35 mg/ml sodium bicarbonate, 10% heat inactivated fetal bovine serum (Byproducts, Guadalajara, México), 50 μg/ml gentamicin (Gibco BRL, Grand Island, NY), 1 mM phenylmethyl-sulfonyl-fluoride (Sigma, St. Louis, MO), 0.2 mM Nα-p-tosyl-L-lysine chloro-methyl ketone (Sigma, St. Louis, MO), and 1 mM leupeptin (Sigma, St. Louis, MO)]. The first drop of perfused hemolymph containing the hemocytes, coming from the tear made on the intersegmentary membrane, was collected on a glass slide to be used on the different assays.

### Effect of AT on hemocyte activation and phagocytic activity

Hemolymph from individual perfused mosquitoes was placed on clean glass slides inside a humid chamber at room temperature for 15 min. After that 2 μl of Grace’s medium (GM) or GM containing AT or NPLP1 (final concentration 10^−7^ M) were added. NPLP1 is a neuropeptide with unknown physiological roles that was used as a negative control [23]. Samples were maintained for additional 15 min in the humid chamber at room temperature, and later fixed in a paraformaldehyde vapor chamber for 30 min. Fixed preparations were air dried, stained for 10 min with 5% Giemsa in PBS and examined in a bright field microscopy. In addition, fresh hemocytes samples (un-fixed) were obtained in similar conditions as described above. Groups of hemocytes from five mosquitoes per treatment (non-treated, AT or NPLP1; by triplicate) were used to evaluate the percentage of hemocytes spreading. A threshold was arbitrary chosen; hemocytes displaying five or more filopodia were considered as “activated” hemocytes (spreading). Samples were analyzed by phase contrast microscopy in a Nikon E-600 microscope (Nikon, Japan).

For phagocytic activity assays, hemocytes after GM, AT (10^−7^ M) or NPLP1 (10^−7^ M) treatments were incubated for 15 min at room temperature with 5 μl of pHrodo Red *Escherichia coli* (8 X 10^6^ /ml in Grace’s medium) (Molecular Probes, Life Technologies, Carlsbad, CA). At the end of the assay, slides were gently rinsed with Grace’s medium and analyzed under phase contrast and epi-fluorescence microscopy (Leica DM 1000, Wetzlar, Germany). Images were recorded with a digital camera (Leica DFC320) using the software Image Manager IM50 from Leica. A minimum of 100 hemocytes were counted from each mosquito sample, and the percentage (%) of phagocytic hemocytes recorded. For each data point, hemocytes were collected from 10 individual mosquitoes. Data are expressed as percentage of phagocytic hemocytes (mean ± SEM). Significant differences were determined with one-way ANOVA followed by Tukey’s test.

### Effect of AT on phagocytic activity of the LSB-AA695BB cell line

The *An*. *albimanus* LSB-AA695BB cell line, originally established from embryos [24], was used to evaluate the ability of Aedes-AT to stimulate phagocytic activity in fibroblast-like mosquito cells. The cell line was cultured in Schneider´s Insect Medium (Caisson Labs, UT) supplemented with 10% heat inactivated fetal bovine serum (Byproducts, Guadalajara, México). Once cultures were confluent, cells were recovered and washed with fresh supplemented medium, and cell concentration was adjusted at 2.5 X 10^5^ cells/ml of supplemented medium. Round glass coverslips (BioCoat 12 mm diameter, Corning Life Sciences, Tewksbury, MA) were placed into each well of 24-well cell culture plates (Corning Costar, NY). One ml of cell suspension was put into each well, and 1 μl of AT or NPLP1 (10^−7^ M final concentration), or 1 μl of sterile water were added. After 30 min at 28°C, the medium was removed, and samples were washed by adding 1 ml of fresh Schneider’s medium. Medium was removed and samples were incubated with 5 X 10^6^ pHrodo Red *Escherichia coli* in 0.5 ml of supplemented Schneider’s medium (20:1, bacteria/cell). Samples were incubated for 30 min at 28°C in dark conditions. Rates of phagocytosis of fluorescent bacteria were evaluated using an epi-fluorescence microscope. A minimum of 100 cells/sample (by triplicate) were counted for each treatment in three independent biological replicates. Data are expressed as percentage of phagocytic hemocytes (Mean ± SEM). Significant differences were determined with one-way ANOVA followed by Tukey’s test.

### AT-QD conjugates binding assays

AT-QD conjugates were diluted in RPMI 1640 culture medium (Gibco BRL, Grand Island, NY). Groups of 10 mosquitoes were inoculated with 0.25 μl of 0.01 μM AT-QD conjugates or non-conjugated QD in RPMI (controls). After 2 h at 28°C, mosquito abdomens were dissected in PBS by cutting along the left lateral membrane, and fixed with 4% formaldehyde in PBS for 2 h at 4°C in dark conditions. Samples were washed three times for 5 min in PBS. Specimens were mounted in slides with 80% glycerol in PBS, and analyzed with an epi-fluorescence microscope.

To label hemocytes inside live mosquitoes we used CM-DiI, a fluorescent dye well suited for monitoring cell movement or location [25]. Briefly, 0.25 ml of a solution of 75 mM CM-DiI (Vybrant CM-DiI Cell-Labeling Solution, Invitrogen) in PBS alone or containing 10^−7^ M of AT were injected into mosquitoes. The solutions were injected immediately after diluting the CM-Dil in PBS or AT/PBS. After CM-DiI injection, mosquitoes were immediately returned to 28°C and 80% relative humidity for an incubation period of 20 min. Then, mosquito abdomens were dissected in PBS by cutting along the left lateral membrane. Preparations were further washed with PBS (3 times, shaking manually the samples between changes for 10–15 seconds), and mounted on microscope slides in a drop of DAPI fluoromount-G medium (EMS, Hatfield, PA). Samples were analyzed in a confocal microscope C2 (Nikon, Japan) attached to an E-600 microscope (Nikon, Japan). Images were processed by using NIS-Elements version 4.2 from Nikon and by Power Point programs. Three-dimensional (3D) reconstructions were obtained from Z-stack data sets. Results are presented as 3D views in a single projection.

### Hemolymph phenoloxidase activity

To evaluate the effect of AT and NPLP1 on hemolymph phenoloxidase (PO) activity, *Ae*. *aegypti* and *An*. *albimanus* adult females were inoculated with 0.25 μl of PBS (controls) or PBS containing AT or NPLP1 (10^−7^ M). Females were kept for 1 h at 28°C. Hemolymph (plasma and hemocytes plus PBS) was obtained by perfusion (as described above), with the first drop (approximately 20 μl) collected in 0.6 ml Eppendorf tubes maintained on ice. The protein concentration in the hemolymph was determined by the Bradford method, using a Protein Assay Kit (Pierce Biotechnology, Rockford, IL). Hemolymph protein concentrations were adjusted to 100 μg/ml with PBS, and PO activity was evaluated by modifying a previously reported method [26]. Briefly, 20 μl of hemolymph and 80 μl of PBS were placed in each well of 96-well plates, immediately, 100 μl of L-dihydroxyphenyl-alanine (L-DOPA, 4 mg/ml in PBS) (Sigma, St. Louis, MO) were added to each well and incubated 1 h at 28°C under dark conditions. The final concentration of hemolymph proteins in the reaction mixture was 10 μg/ml. The absorbance was recorded at 492 nm using a plate reader (Lab systems, Multiskan, Vienna, VA). The effect of peptides on PO activity was expressed in PO units, where one unit represents the amount of enzyme required to produce an increase in absorbance of 0.001/min/mg of protein [26]. Three Groups of 10 mosquitoes were used per treatment, and 3 independent biological replicates were performed. Data are expressed as mean units ± SEM. An unpaired t-test (*p* < 0.05) was performed in samples were PO activity was stimulated.

### Nitric oxide production by cultured guts

*In vitro* gut cultures (midgut, malpighian tubules and hindgut) were prepared as previously reported [27]. Briefly, mosquitos were cold-anesthetized and abdomens were washed with 70% ethanol and air-dried. Dissections were carried out in a drop of PBS containing protease inhibitors (2 mM phenylmethylsulfonyl-fluoride, 0.1 mM Na-p-tosyl-l-lysinechloro-methyl ketone, 1 mM EDTA and 0.1 mg/ml leupeptin; all from Sigma, St. Louis, MO). Four guts per well were collected in a 96-well culture plate (Nalgene Nunc Co., Naperville, IL) containing 200 μl of RPMI pH 8.3 without phenol red, supplemented with 10% inactivated fetal bovine serum (Byproducts, Guadalajara, México). An antibiotic-antimycotic mixture (100 units/ml of penicillin, 100 μg/ml of streptomycin and 0.25 μg/ml of amphotericin B; Gibco BRL, Grand Island, NY) was added.

To evaluate the effect of AT on nitric oxide (NO) production, *An*. *albimanus* guts were treated *in vitro* with either: A) 2 μl of AT (10^−7^ final concentration), B) 2 μl of phorbol 12-myristate 13-acetate (10 ng/ml final concentration), an inducer of nitric oxide production in invertebrate hemocytes [28], (PMA, Sigma, St. Louis, MO), C) 20 μl of lipopolysaccharide, an strong elicitor of immune responses, (100 μg/ml final concentration) (LPS from *Escherichia coli* 0111: B4, Sigma, St. Louis, MO) [29], or D) 2 μl of sterile water. After a 24 h *in vitro* incubation at 22°C with any of the 4 treatments, guts were removed and NO concentrations were determined in the culture medium by the Griess reaction [30], as previously reported by Herrera-Ortiz et al. [27]. Briefly, fifty μl of each culture medium were mixed with 50 μl of 1% sulfanilamide and 50 μl of 0.1% naphthylethylenediamine (Sigma, St. Louis, MO), and incubated for 10 min at room temperature. The absorbance was recorded at 540 nm using a plate reader (Lab systems, Multiskan, Vienna, VA). NO was quantified using a NaNO_2_ standard reference curve (1–100 μM). The results are expressed as mean concentration of nitrites ± SEM from three independent experiments. Significant differences were determined with one-way ANOVA followed by Tukey’s test.

### Expression of antimicrobial peptide genes

Gut *in vitro* cultures were stimulated with AT or LPS as described for the NO assays. After 24 h at 22°C, total RNA was isolated using the Quick-RNA Mini Prep kit (Zymo Research, Irvine, CA). Briefly, guts were transferred to a microtube containing 100 μl of Buffer ZR RNA and homogenized with a pestle. Homogenates were transferred into a Zymo-Spin Column TM and centrifuged at 12,000 rpm for 1 min followed by several washing steps. The RNA was eluted with 30 μl of DEPC-H2O, and its integrity evaluated in 1.5% agarose gel electrophoresis. cDNA was synthesized by reverse transcription with the GeneAmp® RNA PCR kit (Applied Biosystems, CA), using 1 μg RNA, 100 ng oligonucleotide dT, and 200 U of reverse transcriptase RNase H SuperScript II (Gibco BRL, Grand Island, NY). The reaction mixture also contained 50 mM Tris-HCl pH 8.3, 75 mM KCl, 3 mM MgCl_2_ and 10 mM DTT. Reverse transcription was performed in a thermocycler GeneAmp PCR system 2400 (Applied Biosystems), with the following run conditions: 15 min at 42°C, 5 min at 99°C and finally 5 min at 5°C. The cDNA's generated were stored at 4°C for further processing.

Quantitative real time PCR (qPCR) was performed with 2.5 μl of cDNA and the Syber Green I Kit (Applied Biosystems, CA). We used the previously reported primers [31]: 1) gambicin (AGAP008645) RT_Gam_F (CGTGCGATGGTCAGACGAT) and RT_Gam_R (CGCCGCGTTCACAAGAA), 2) attacin (AGAP005620) Atta_F (CGC TAC AAA GGC AAG ATG AAC) and Atta_R (TGT TTC CGC TCG CAC TCT TC), and 3) cecropin (AGAP000694) Cec3_F (GAAATTGGCAAACGACGTGAA) and Cec3_R (GCGATGCTAAAAGACTAAGGGC). As an internal control, a fragment of actin was amplified using the following primers RT_ActU_R (CGA TCC ACT TGC AGA GCC AGT) and RT_Act3.2_F (TAC GCC AAC ATT GTC ATG TCC). The amplification and detection of specific products was performed on an ABI Prism 7900 HT real-time PCR system (Applied Biosystems, CA), using the following conditions: 1 cycle at 48°C for 10 min, 1 cycle at 95°C for 10 min, 40 cycles at 95°C for 15 s and 1 cycle at 60°C for 1 min. The fold changes in expression were calculated using the comparative “delta delta Ct” (ΔΔCt) method against the untreated control guts. Two independent experiments using three replicates per sample were performed, and the data represent the average fold-changes relative to control groups (sterile water). The amplification efficiency was similar between the test and control genes [31, 32].

### Statistical analyses

Statistical analyses of the data were performed using GraphPad Prism version 3.0 for Windows (Graph-Pad Software, San Diego, CA).

## Results

### Cellular responses: AT induced hemocyte phenotypic changes and stimulated bacterial phagocytic activity

**Fig 1:**
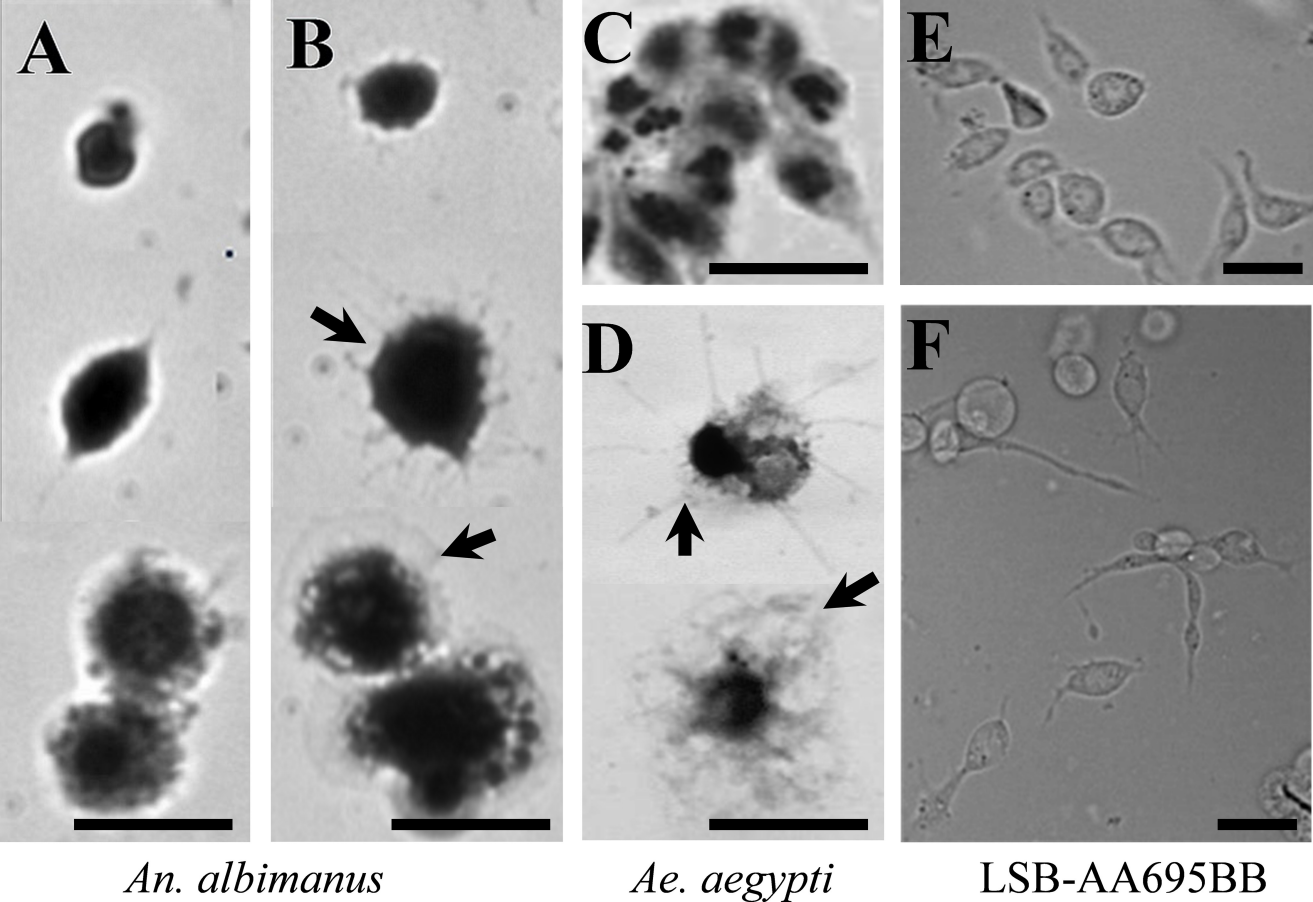
AT induced phenotypic changes on mosquito hemocytes and LSB-AA695BB cells. Hemocytes from *An*. *albimanus* (**A-B**) and *Ae*. *aegypti* (**C-D**) were obtained by perfusion and incubated in a humid chamber with: Grace´s medium alone (**A, C**) or AT (10^−7^ M) (**B, D**). After 30 minutes, samples were fixed, stained with Giemsa and observed by bright-field microscopy. LSB-AA695BB cells were grown in a glass cover-slide, and then incubated 30 min in: Schneider´s medium alone (**E**) or containing AT (10^−7^ M) (**F**). Samples were observed by phase contrast microcopy. Hemocyte spreading (arrows). Scale bars: 5μm.

*Aedes*-AT triggered marked morphological changes in hemocytes from *Ae*. *aegypti* and *An*. *albimanus*, as well as on an newly-described immuno-responsive cell line from *An*. *albimanus*. The typical morphology of *An*. *albimanus* hemocytes after 30 minutes *in vitro* culture incubation is shown in Fig 1A and supplemental S1 Fig and S2 Fig. Morphological changes observed in hemocytes treated with AT (arrows in Fig 1B, 1D, S1 Fig and S2 Fig), resembled cell spreading previously described in *An*. *gambiae* hemocytes [33, 34]. Similar results were observed with *Ae*. *aegypti* hemocytes (Fig 1C, 1D, S1 and S2 Figs). In addition, we evaluated the morphological changes induced by AT on *An*. *albimanus* LSB-AA695BB cells. This is a fibroblast-like cell line established from mosquito embryos [24]. The cell line also displayed strong morphological changes (spreading) in the presence of AT (Fig 1E and 1F). The NPLP1 peptide did not induce morphological changes on hemocytes of both mosquito species, as well as on the LSB-AA695BB cell line. All the samples treated with this peptide showed similar morphology than the control samples (data not showed).

**Fig 2:**
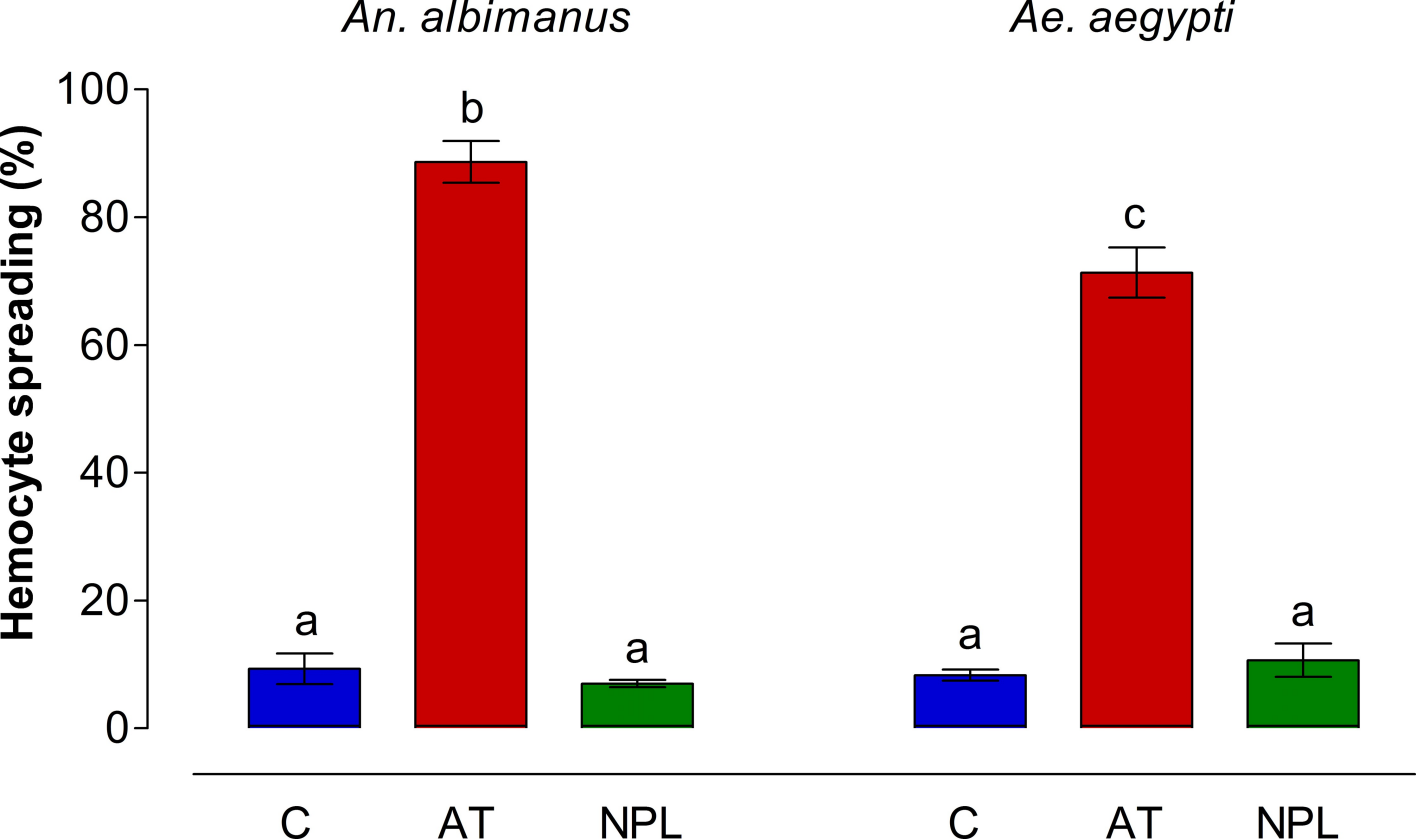
Hemocyte spreading percentages in *An*. *albimanus* and *Ae*. *aegypti*. Mosquito hemocytes were obtained by perfusion, and incubated in a humid chamber with either Grace´s medium alone (C), AT (10^−7^ M) or NPLP1 (NPL, 10^−7^ M). After incubation, samples were analyzed by contrast phase microscopy and hemocytes showing spreading were recorded. Each hemocyte sample from groups of five mosquito per treatment was individually analyzed. Three independent experiments were performed. Data are expressed as percentage of spreading (Mean ± SEM). Significant differences were determined with one-way ANOVA followed by Tukey’s test (*p <* 0.001). Different letters on the top each bar are significantly different.

The percentage of hemocytes showing spreading was evaluated in perfused hemolymph samples from groups of five mosquitoes per treatment (in three independent experiments). The number of hemocytes showing five or more filopodia (activated) was significantly higher (*p <* 0.001) in samples treated with AT than in NPL (NPLP1) or control (non-treated) samples (Fig 2).

**Fig 3:**
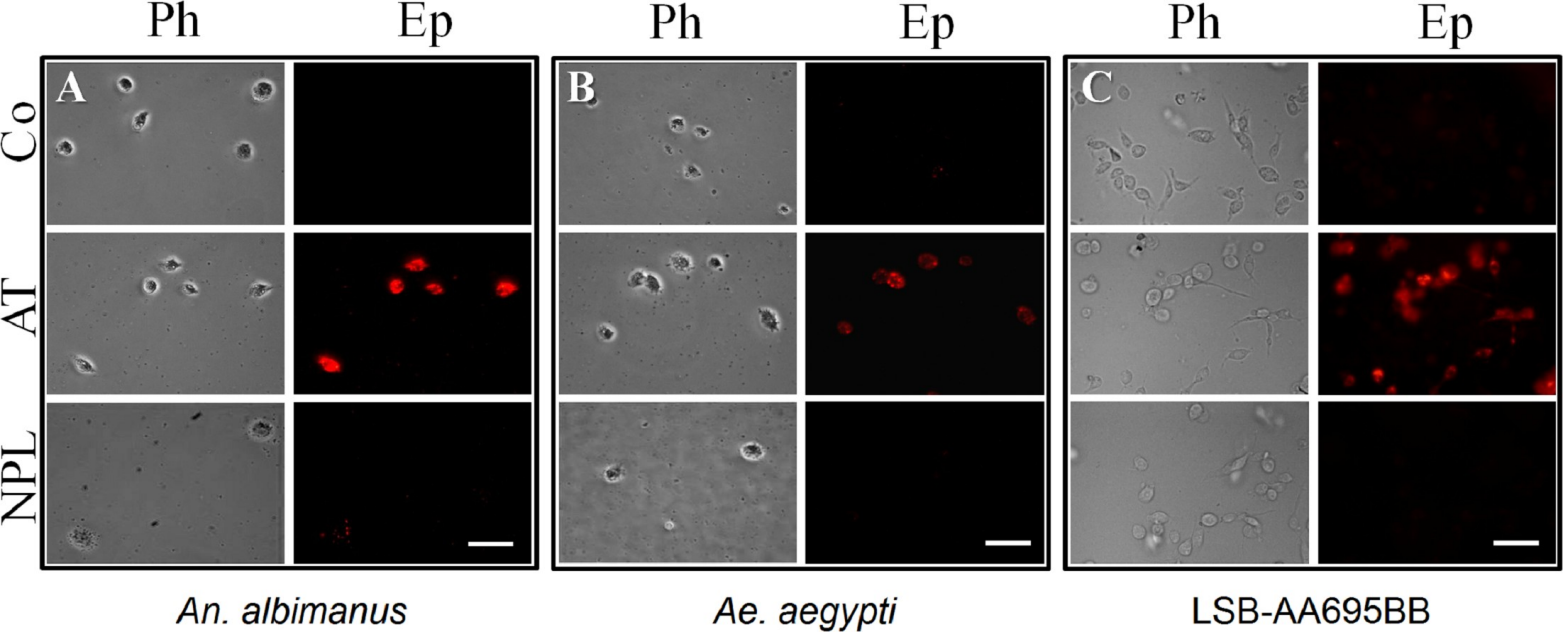
Allatotropin (AT) stimulates bacteria phagocytosis by mosquito hemocytes and LSB-AA695BB cells. *An*. *albimanus* (**A**) and *Ae*. *aegypti* (**B**) hemocytes were obtained by perfusion and incubated alone (Co) or with 10^−7^ M of Aedes-AT (AT) or NPLP1 (NPL), before adding pHrodo *E*. *coli* bacteria. LSB-AA695BB cells (**C**) were grown in a glass cover-slide, and then incubated in Schneider´s medium alone (Co) or with 10^−7^ M of Aedes-AT (AT) or NPLP1 (NPL), before adding pHrodo *E*. *coli* bacteria. Ph: phase contrast microscopy; Ep: epi-fluorescence microscopy. Scale bars, 10 μm.

**Fig 4:**
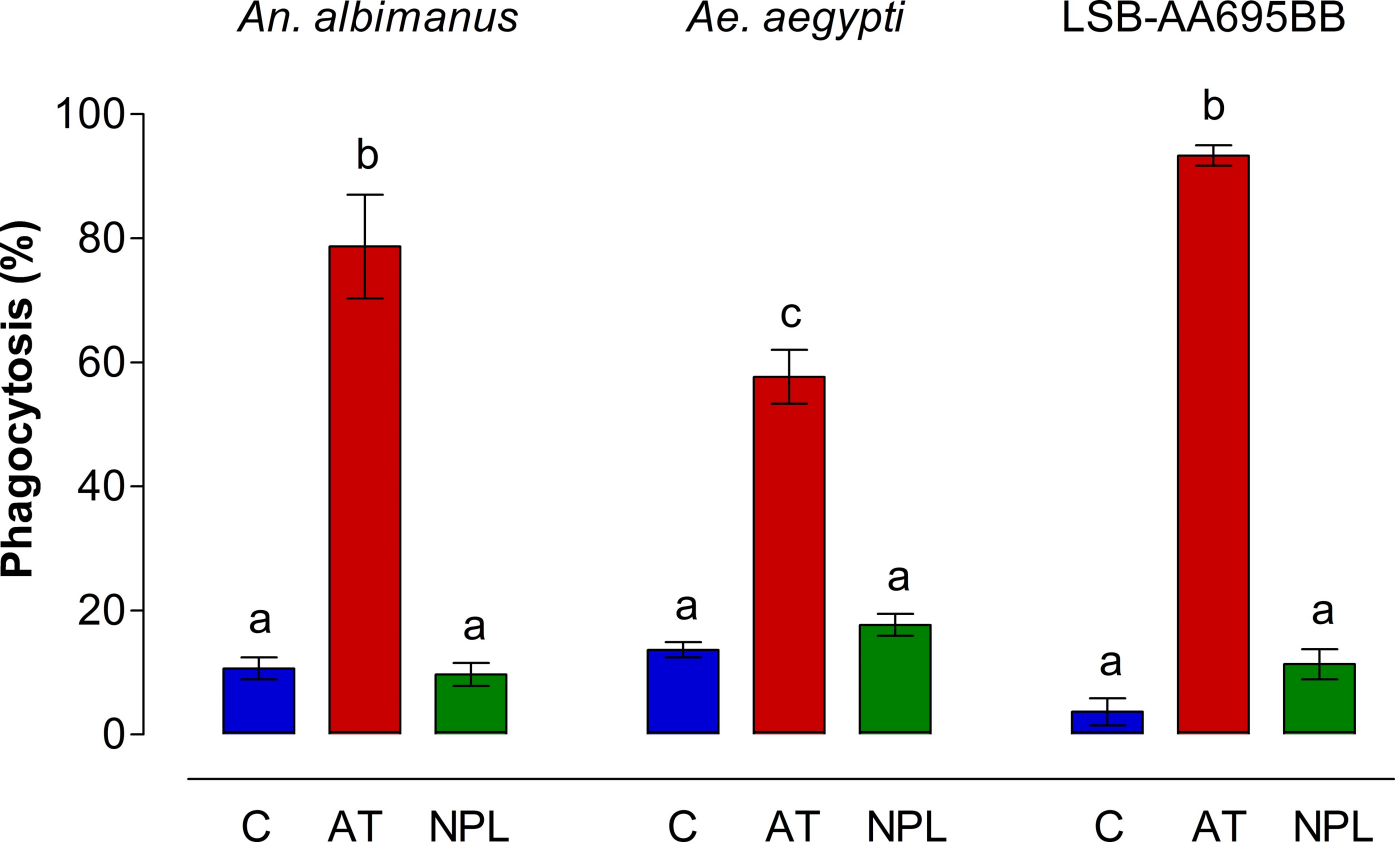
AT increases phagocytic activity by mosquito hemocytes and LSB-AA695BB cells. Number of cells displaying fluorescent phagocytic vacuoles in hemocytes and LSB-AA695BB after incubation with medium (C) or with 10^−7^ M of Aedes-AT (AT) or NPLP1 (NPL). A minimum of 100 cells/sample (by triplicate) were counted for each treatment in three independent biological replicates. Data are expressed as percentage of phagocytic hemocytes (Mean ± SEM). Significant differences were determined with one-way ANOVA followed by Tukey’s test (*p <* 0.05). Different letter on the top each bar are significantly different.

In addition to these morphological changes, we evaluated the effect of AT on phagocytic activity of fluorescent bacteria by hemocytes isolated from the 2 mosquito species, as well as by *An*. *albimanus* LSB-AA695BB cells (Fig 3). Because pHrodo bacteria produce fluorescence only when they are inside phagocytic vacuoles, they are ideal to evaluate phagocytic activity. *An*. *albimanus* (Fig 3A) and *Ae*. *aegypti* (Fig 3B) hemocytes obtained by perfusion and exposed to AT were highly phagocytic compared with those incubated with NPLP1 or control samples. Allatotropin also induced strong phagocytic activity in the *An*. *albimanus* cell line; most LSB-AA695BB cells pre-incubated with AT, were able to engulf pHrodo bacteria after 30 min of treatment (Fig 3C). No attempts to compare the number of phagocytic vacuoles in individual cells between different treatments were performed, and the presence of a single fluorescent vacuole was enough to be considered as a positive phagocytic cell. AT induced significant increases in the number of phagocytic hemocytes of both mosquito species, as well as phagocytic LSB-AA695BB cells, when compared with control samples and NPLP1 treated samples (Fig 4).

**Fig 5:**
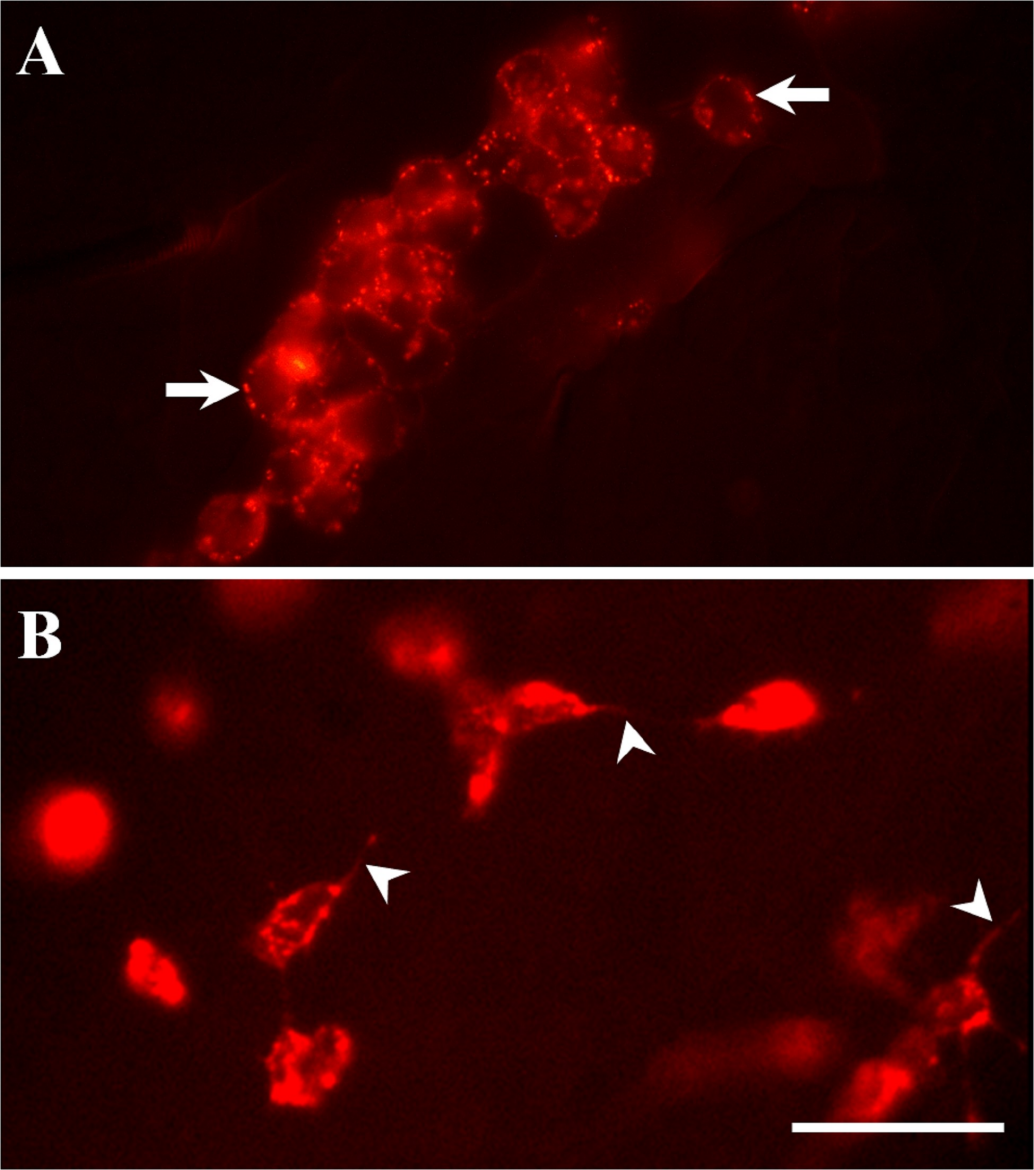
Allatotropin-QD conjugates attached to mosquito hemocytes *in vivo*. AT-QD conjugates were inoculated into the hemocoel of *An*. *albimanus* (A) and *Ae*. *aegypti* (B). After 2 h, abdomens were dissected, fixed and analyzed by epi-fluorescence microscopy. The label showed a pattern of micro vesicular distribution, and was more evident on hemocyte surfaces (arrows) Cells with long pseudopodia projections (arrow heads) were observed in hemocytes attached to abdominal pleural membranes. Scale bar, 10 μm.

To assess if AT-QD conjugates bind to hemocytes *in vivo*, we inoculated AT-QD conjugates into *An*. *albimanus* and *Ae*. *aegypti* hemocoel. Only hemocytes were specifically labeled by the AT-QD conjugates in both mosquito species (Fig 5, S3 and S4A Figs). The label showed a pattern of micro vesicular distribution, and was more evident on hemocyte surfaces. Hemocytes were frequently seen aggregated (Fig 5A, S6 and S7 Figs), and some the labeled hemocytes exhibited long pseudopodia projections, which are typical of activated cells (Fig 5B). Most labeled hemocytes were attached to tissues, particularly to lateral pleural membranes, inter-segmentary membranes, dorsal vessel, traqueoles, midgut and fat body (Fig 5 and S3–S7 Figs). Control samples inoculated with streptavidin-QD alone (non AT-conjugated), showed a diffuse signal at the surface of most tissues, with no specific cell type labeled (S4B Fig). A schematic drawing is provided (S5 Fig), to indicate the specific abdominal areas showing labeled AT-QD in figures S3 and S4 Figs.

### Humoral responses: AT induced phenoloxidase activity, nitric oxide generation and transcription of antibacterial peptides

**Fig 6:**
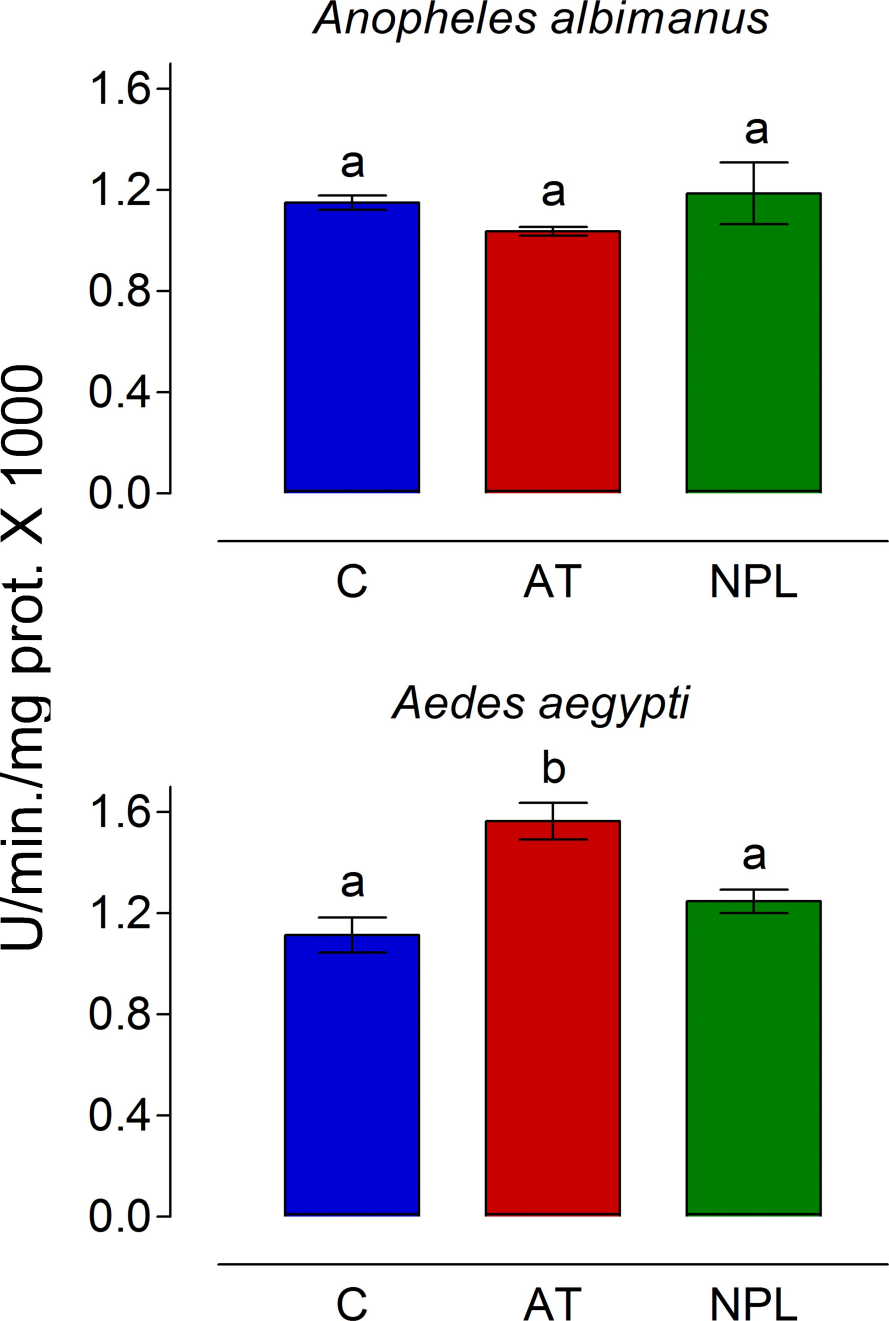
AT injection increased phenoloxidase (PO) activity in *Ae*. *aegypti* hemolymph. Mosquitoes were inoculated with 0.25 μl of PBS alone (C) or with 10^−7^ M of Aedes-AT (AT) or NPLP1 (NPL). After 1 h, hemolymph was obtained by perfusion and PO activity was evaluated. PO activity units represent the amount of enzyme required to produce an increase in absorbance of 0.001/min/mg of protein. Bars represent the means ± SEM of three independent replicates of groups of 10 mosquitoes. Significant differences were determined with one-way ANOVA followed by Tukey’s test (*p <* 0.05). Different letter on the top each bar, are significantly different.

To analyze the effect of AT on hemolymph phenoloxidase (PO) activity, mosquitoes were inoculated with 0.25 μl of AT, NPLP1 (both 10^−7^ M in PBS) or PBS alone. The hemolymph obtained by perfusion was incubated with L-DOPA for 1 h at 28°C under dark conditions and the absorbance was recorded at 492 nm. Phenoloxidase activity was not modified by AT inoculation in *An*. *albimanus* (Fig 6). However, samples from *Ae*. *aegypti* inoculated with AT showed a significant increase in PO activity as compared with NPLP1 or PBS-inoculated samples (Fig 6).

**Fig 7:**
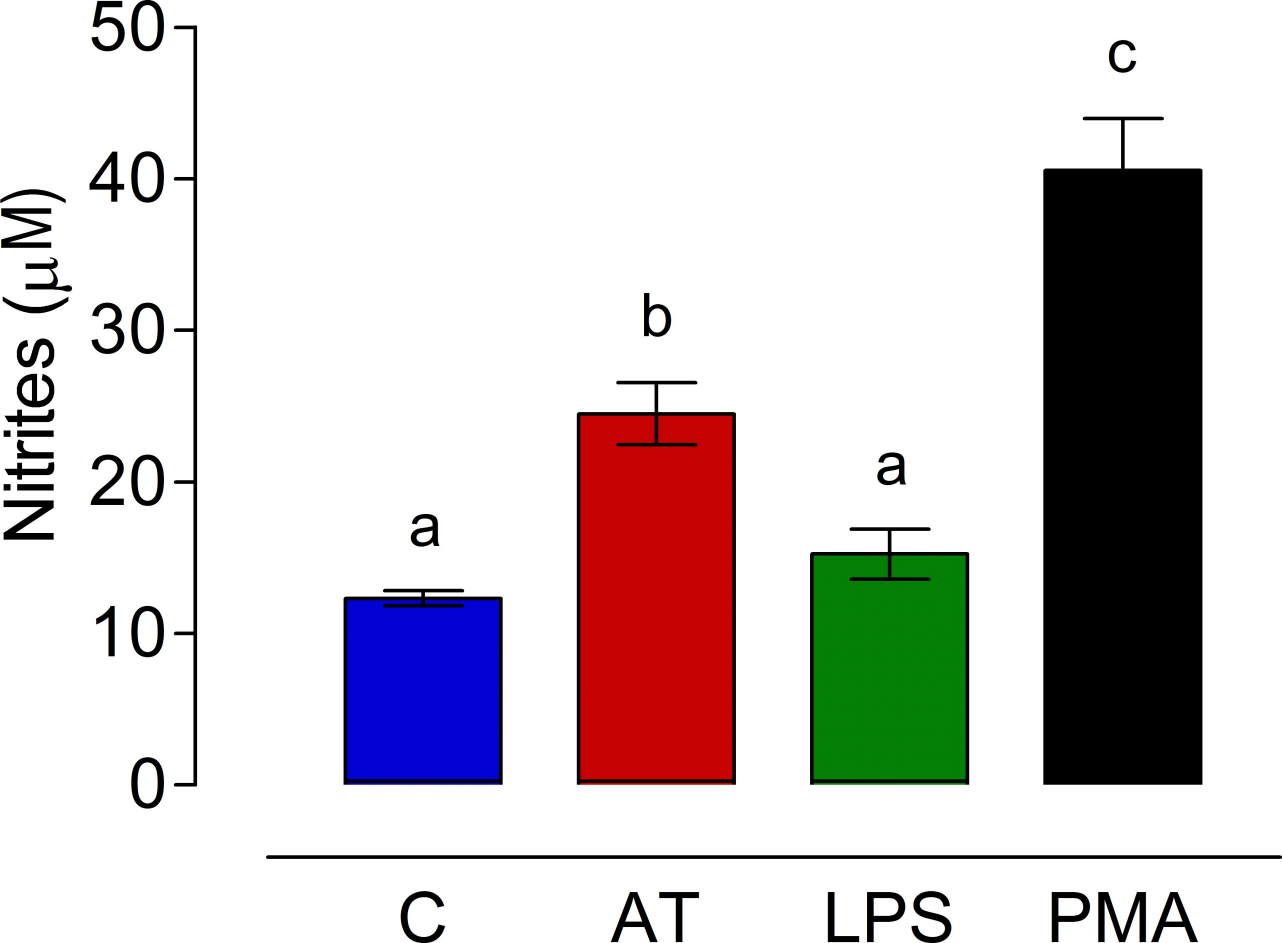
AT induced nitric oxide (NO) generation. *An*. *albimanus* guts were incubated for 24h in medium alone (C) or with 10^−7^ M of Aedes-AT (AT), *E*. *coli* lipopolysaccharide (LPS) (100 μg/ml) or phorbol 12-myristate 13-acetate (PMA) (10 ng/ml). NO concentration was evaluated in the culture medium by the Griess reaction. Results are expressed as mean concentration of nitrites ± SEM of three independent experiments. Significant differences were determined with one-way ANOVA followed by Tukey’s test (*p <* 0.05).

Allatotropin also stimulated nitric oxide (NO) generation by *An*. *albimanus* guts. After 24h in culture, guts treated with AT displayed a significant increase in NO generation compared with those treated with sterile water or LPS (Fig 7). PMA induced the strongest NO production, and it was significant different from the other treatments (Fig 7).

**Fig 8:**
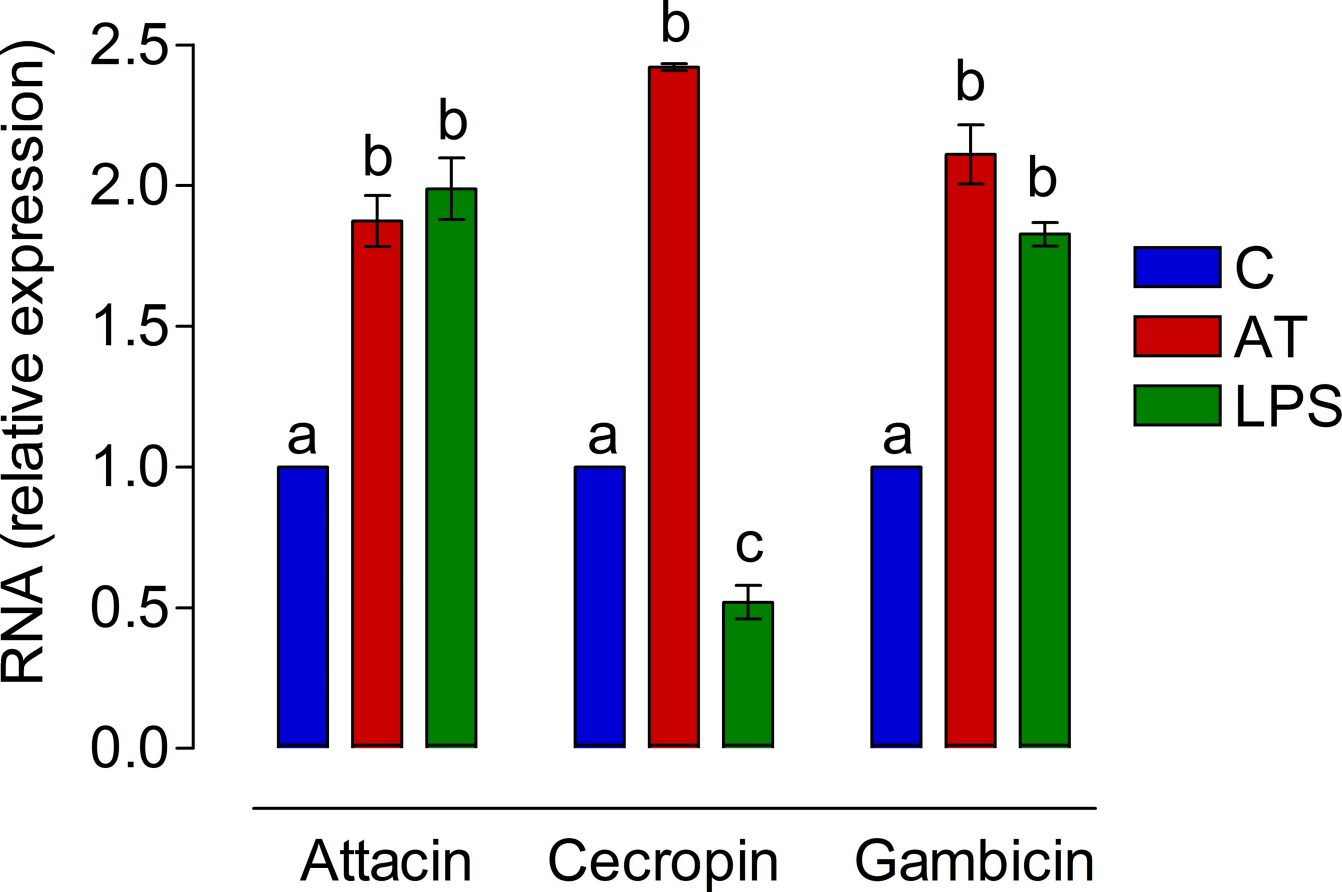
AT induced antibacterial gene expression in *An*. *albimanus* cultured guts. *An*. *albimanus* guts were incubated for 24h in medium alone (C) or with 10^−7^ M of Aedes-AT (AT) or E. *coli* lipopolysaccharide (LPS) (100 μg/ml). Relative expression of gambicin, attacin, and cecropin genes were measured by qPCR. Two independent experiments using three replicates per sample were performed and RNA (relative expression) represents the average fold-change relative to the control group (X_T_/X_C_). Significant differences were determined with one-way ANOVA followed by Tukey’s test (*p <* 0.05).

In addition, *in vitro* AT and LPS treatment induced the expression of gambicin, attacin, and cecropin genes in *An*. *albimanus* guts. The relative production of the three antimicrobial peptide mRNA was increased almost 2-fold in samples treated with AT (Fig 8). Interestingly, treatment with LPS also increased the expression of attacin and gambicin, but not the expression of cecropin mRNA (Fig 8).

## Discussion

Insects have several types of hemocytes that are distinguished by morphology, molecular and antigenic markers, and function. Hemocytes from *An*. *albimanus* were originally classified as granular cells, prohemocytes, and plasmatocytes [22]. Hillyer and Strand recently described that *Ae*. *aegypti* and *An*. *gambiae* contain three populations of hemocytes: granulocytes, oenocytoids and prohemocytes [12], with granulocytes been equivalent to those originally described as plasmatocytes in *An*. *albimanus*. In the present study, we did not implement a comparison between hemocyte types in both mosquito species; instead, we will refer to them in general as “hemocytes”.

The first indication of a possible role of allatotropic peptides in the activity of insect hemocytes was reported by Skinner et al. [35]; their studies described that allatostatin-A (AST-A) was present in granular hemocytes of the cockroach *Diploptera punctata*, leading them to suggest that AST-A could have new functions, such as participation in cell-mediated immune responses. In mammals, distinct morphological changes occur during a cell-activating immune response, and the macrophage spreading assay has been widely used to analyze cell activation [36]. Macrophage activation involves different cytokines, with interferon-γ and tumor necrosis factor playing important roles [37]. Mosquito hemocytes are also able to attach and spread when cultured *in vitro*, and the spreading is faster if they are “primed” (activated) with an immune elicitor [22]. Immune-activated hemocytes might bind to surfaces through adhesion factors and/or cell surface receptors. The activation leads to spreading of filopodia of granulocytes and pseudopodia/lamellipodia of plasmatocytes [38]. Recently, some studies have described important changes in the number, aggregation properties, size, granularity, and molecular activation markers (TEP1, pERK and PPO6) in hemocytes of *An*. *gambiae* after a blood-meal [33, 34].

In *An*. *albimanus* and *Ae*. *aegypti* hemocytes, as well as in the immune-responsive cell line, the treatment with AT induced drastic morphological changes that suggest an immune-like activation process. These changes included generation of numerous pseudopodia and filopodia. The increased adherence of hemocytes to different tissues observed in the present study could be related to changes on surface carbohydrate moieties as described in *An*. *gambiae* [33]. Moreover, it is very interesting that the increased PO activity observed in *Ae*. *aegypti* was also described in *An*. *gambiae* as an activation marker after a blood meal [33, 34].

Molecular interactions between AT and hemocytes were confirmed by the specific label of hemocytes by AT-QD conjugates. The finding that most labeled-hemocytes were attached to tissues, such as fat body, tracheoles, pleural membranes and midgut (S3–S7 Figs), emphasizes the ability of AT-stimulated hemocytes to spread and attach to surfaces. To visualize better the effect of AT on hemocytes *in vivo*, we used the lipophilic fluorochrome chloromethyl-dialkylcarbocyanine (CM-dil) to achieve hemocyte-specific staining. Injection of AT into the mosquito hemocoel increased the amount of hemocytes strongly attached to abdominal tissues, including the heart (S8 and S9 Figs, S1 Video). Previous studies reported that after an infection, *An*. *gambiae*, hemocytes were preferentially aggregated in specific regions of the heart, emphasizing the role of the circulatory system in the hemocyte-mediated immune response [39, 40]. The hemocyte aggregation to the tracheal system was previously reported in adults and larvae of *An*. *gambiae* [41, 42].

Although, there is a widespread distribution of AT-receptor in mosquito tissues, including CA [11], only hemocytes were recognized by the AT-conjugates. This is not surprising because of the highly selective basal lamina that separates most tissues from the hemolymph. In *An*. *gambiae*, the hemocyte-specific staining with CM-dil was also attributed to the inability to cross the basal lamina of other tissues [39]. The insect basal lamina is composed of an amorphous association of proteins and glycosaminoglycan that functions as a primary barrier to the movement of macromolecules between tissues and the hemolymph [43]. The largest size of macromolecules that cross the basal lamina have been reported to be 5–8 nm in tissues of *Culex tarsalis* [44], and 11 nm in *Ae*. *aegypti* [45]. In the lepidopteran *Calpodes ethlius*, basal lamina from organs involved in the import/export of large hemolymph proteins (e.g. fat body or pericardial cells) were permeable to 15 nm particles, whereas other organs excluded particles larger than 6 nm [43]. Our QD-AT conjugates have an average diameter of 30–32 nm, making it difficult to cross the basal lamina of tissues.

Phagocytosis is an evolutionarily conserved process that in insects is hemocyte-mediated. Microorganisms are recognized by pattern recognition receptors (PRRs), both soluble in the hemolymph (working as opsonins) or fixed on the surface of hemocytes. Microorganisms are internalized into a phagosome that transforms into a phagolysosoma, where they are digested. In mosquitoes, several factors opsonize microorganisms and promotes phagocytosis, among them are: TEP1 (thioester containing protein 1), *Anopheles-Plasmodium*-responsive leucine-rich repeat 1 (APL1), fibrinogen-related protein 8 (FBN8), and down syndrome cell adhesion molecule (DSCAM) [46–48]. Interestingly, some of these proteins not only works as opsonins, but also have roles as stabilizing factors of opsonizing complexes (e.g. LRIM1 and APL1) [49]. AT might be part of a receptor-mediated process that “activate” or “prime” the cell for phagocytosis, by inducing hemocyte morphological changes (spreading). Our experimental results revealed that hemocytes were not only “activated” by AT, but also phagocytic activity was increased, as evidenced by pHrodo-bacteria engulfment. Mosquito hemocytes are able to initiate phagocytosis of bacteria *in vitro* without additional stimuli [22, 50]; however, the process is relatively slow, taking at least 30–60 min to initiate [22]. In the present studies, some of the control and NPLP1-treated hemocytes were seen with engulfed pHrodo-bacteria only after 40 min incubations (data not showed); however, treatment with AT elicited a faster phagocytic response (15 min) in 60–80% of the hemocytes. In addition, we observed that normally LSB-AA695BB cells have a limited phagocytic capacity (generally less than 30% of phagocytic cells are observed after 2 h of interaction with bacteria), but pre-incubation with AT elicited phagocytic activity in more than 90% of the cells.

Most pleiotropic functions of AT, including cardioacceleratory stimulation and induction of hindgut and oviducts contractions, are associated with intracellular calcium changes. CA activity is stimulated by Ca^2+^ ionophores, while membrane-permeable chelators antagonize the stimulatory effects of AT, suggesting that AT may enhance CA activity by increasing intracellular Ca^2+^ concentrations [51]. It is possible that in mosquito hemocytes, AT elicits morphological changes and cell activation (including phagocytosis) through a similar mechanism of calcium modulation. In mammal macrophages it is recognized the importance of increasing calcium concentration for the changes in the cytoskeleton, essential for cell spreading, motility and phagocytosis [52].

AT-like peptides have been identified in different invertebrate groups including Arthropoda, Mollusca and Annelida (for review see [53]). The extensive distribution of AT immunoreactivity in *Ae*. *aegypti* and *An*. *albimanus* [8], together with the widespread expression of AT receptor mRNA in different mosquito tissues [11], suggest that AT may have additional biological activities in mosquitoes besides its stimulatory role on CA activity. In the present work we described that AT-QD conjugates labeled hemocytes *in vivo* in both *Ae*. *aegypti* and *An*. *albimanus*, suggesting molecular interactions of AT with hemocyte surface receptors. AT-induced phenotypic changes included spreading, attachment and increased phagocytic activity. In addition, phenoloxidase activity in hemolymph was also augmented in *Ae*. *aegypti* mosquitoes treated with AT but not in *An*. *albimanus*, suggesting differences in the AT-dependent immune activation in the two species. Finally, two important insect immune markers, generation of nitric oxide and the expression of messengers for antimicrobial peptides were also induced by AT in *An*. *albimanus* guts. In summary, we provided experimental evidence that treatment *in vitro* with AT elicits a wide range of cellular and humoral immune responses by *An*. *albimanus* and *Ae*. *aegypti* hemocytes and guts, as well as by an immuno-responsive mosquito cell line. The physiological relevance of these AT-induced responses *in vivo* remain to be tested; the possibility that AT is released into the hemolymph and exerts similar effects on hemocytes and guts *in vivo* cannot be ruled out at this moment. AST-A has been shown to be present in the cell-free fraction of cockroach hemolymph (plasma), where it binds to hemocytes [35]. The mosquito gut is the first interface where host-environment crosstalk takes place. In addition to digestive and excretory functions, the gut cells mediate interactions of the mosquito with a diverse community of microbes, such as viruses, bacteria, fungi, and parasites. The mosquito gut is therefore an important arm of the mosquito immune response [54], and AT immunoreactive cells are present in every segment of the mosquito ventral ganglia, with immunostained projections innervating the gut [8]. In summary, the physiological relevance of these AT-induced responses remains to be tested *in vivo*, both in normal conditions and during the infection with pathogens. To elucidate some of those questions should impulse new directions for this area of research.

## Supporting information

S1 Fig Morphological changes induced in mosquito hemocytes by AT. Hemocytes were obtained by perfusion and incubated in Grace’s medium alone (control) or containing AT (final concentration 10^−7^ M). After a 15 min incubation, samples were examined with a 20X objective by phase contrast microscopy. Morphological changes were evident in hemocytes of both mosquitoes when treated with AT. Scale bar: 40 μm.
(JPG)

S2 Fig Morphological changes induced in mosquito hemocytes by AT. Hemocytes were obtained by perfusion and incubated in Grace’s medium alone (control) or containing AT (final concentration 10^−7^ M). After a 15 min incubation, samples were examined with a 100X objective by phase contrast microscopy. A threshold was arbitrary chosen; hemocytes displaying five or more filopodia were considered as “activated” (spreading). Samples were analyzed by phase contrast microscopy in a Nikon E-600 microscope (Nikon, Japan). Each panel in the figure is a composition of different fields in the samples. The presence of variable number of filopodia were evident in both mosquito hemocytes treated with AT. Scale bars: 20 μm.
(JPG)

S3 Fig AT-QD-conjugates recognized *An*. *albimanus* hemocytes *in vivo*. AT-QD conjugates were injected into the hemocoel and incubated for 2 h. Abdomens were dissected and analyzed by epi-fluorescence microscopy. Conjugates recognized *An*. *albimanus* hemocytes (red) distributed along the mosquito abdomen. Labeled hemocytes (arrows) were mainly accumulated on the borders of each abdominal segment (dashed boxes indicates the 2^th^ (a) and 3^th^ (b) abdominal segments). Hemocytes were attached to fat body, tracheoles (arrowheads) and lateral (asterisks) and inter-segmentary (arrows) pleural membranes. Dot-arrows point the abdomen anterior side. DV, dorsal vessel. Scale bar: 100 μm.
(JPG)

S4 Fig AT-QD-conjugates recognized *Ae*. *aegypti* hemocytes *in vivo*. AT-QD conjugates **(A)** or QD-streptavidin alone **(B)** were injected into the hemocoel and incubated for 2 h. Abdomens were dissected and analyzed by epi-fluorescence microscopy. AT-QD conjugates recognized *Ae*. *aegypti* hemocytes (red) in the mosquito abdomen. Labeled hemocytes (arrow heads) were mainly accumulated in the borders of each abdominal segment. Hemocytes were also attached to fat body, tracheoles and lateral pleural membranes (asterisks). QD-streptavidin alone produced a diffuse background with some accumulations in non-specific regions. Dashed lines indicate section of the 5^th^ dorsal and ventral abdominal segment. Dot-arrows point the abdomen anterior side. d, dorsal area; v, ventral area. *Lateral pleural membrane of the abdomen. Scale bars: 100 μm.
(JPG)

S5 Fig Representation of a mosquito abdomen, showing the areas analyzed for AT-QD conjugates interactions. (A) Dashed box green lines correspond to the area showed in figure S3. (B) Dashed green lines correspond to the area showed in figure S4. The anterior part of the drawing represents the first abdominal segment. Arrowheads mark the area where the last (8^th^) abdominal segment was detached.
(JPG)

S6 Fig AT-QD conjugates recognized hemocytes aggregated on the midgut surface. AT-QD conjugates were injected into the hemocoel and incubated for 2 h. Abdomens were dissected and analyzed by epi-fluorescence microscopy. *An*. *albimanus* (**A**) and *Ae*. *aegypti* (**B**) hemocytes attached to the midgut (mg) surface were recognized by the conjugates (red fluorescence). A and B: left, light microscopy; middle, epi-fluorescence microcopy; right, merged image. Scale bar: 20 μm.
(TIF)

S7 Fig AT-QD conjugates recognized mosquito hemocytes aggregated on abdominal fat body surfaces. AT-QD conjugates were injected into the hemocoel and incubated for 2 h. Abdomens were dissected and analyzed by epi-fluorescence microscopy. *An*. *albimanus* (**A**) and *Ae*. *aegypti* (**B**) hemocytes are displayed attached to fat body cells (fb) (red fluorescence). Cell nuclei are stained with DAPI (blue). Scale bar: 20 μm.
(TIF)

S8 Fig Hemocyte distribution in abdomens of mosquitoes treated with AT *in vivo*. Mosquitoes were injected with CM-Dil alone (A, C) or mixed with 10^−7^ M of AT (B, D). After incubation, abdomens were dissected and analyzed by confocal microscopy and the images are presented as 3D views in a single projection. AT treatment increased the amount of hemocytes attached to abdominal tissues (red dots). In each panel, abdomens are orientated with the anterior side to the right. Cell nuclei are stained with DAPI (blue). Arrows: heart position. *Ventral area; **Dorsal area. Scale bar: 1 mm.
(JPG)

S9 Fig Hemocyte distribution in abdomens of mosquitoes treated with AT *in vivo*. Mosquitoes were injected with CM-Dil alone or mixed with 10^−7^ M of AT (CM-Dil/AT). After incubation, abdomens were dissected and analyzed by confocal microscopy and the images are presented as 3D views in a single projection. AT treatment increased the amount of hemocytes (red) attached to abdominal tissues, including the heart (arrow in A). Abdomens are orientated with the anterior side to the right in panel A. (B) Detail of *in vivo* morphological changes in hemocytes after AT treatment. Cell nuclei are stained with DAPI (blue). Arrows: heart position. *Ventral area; **Dorsal area. Scale bar: 1 mm.
(JPG)

S1 Video Hemocyte distribution in mosquito heart (5^th^ abdominal section) treated with AT *in vivo*. Mosquitoes were injected with CM-Dil alone or mixed with 10^−7^ M of AT (CM-Dil/AT). After incubation, abdomen was dissected and analyzed by confocal microscopy with 40X objective, and the video is the three-dimensional (3D) reconstruction obtained from Z-stack data set. Large cell nuclei stained with DAPI (blue) corresponds to pericardial cells aligned laterally to the heart. Hemocytes (red) are preferably aggregated to periostial regions of the heart.
(MP4)
