# Supplementary figures and images for: Allatotropin: A pleiotropic neuropeptide that elicits mosquito immune responses
Source: PLoS One. 2017 Apr 20;12(4):e0175759. doi: 10.1371/journal.pone.0175759 (PMC5398552; doi:10.1371/journal.pone.0175759)

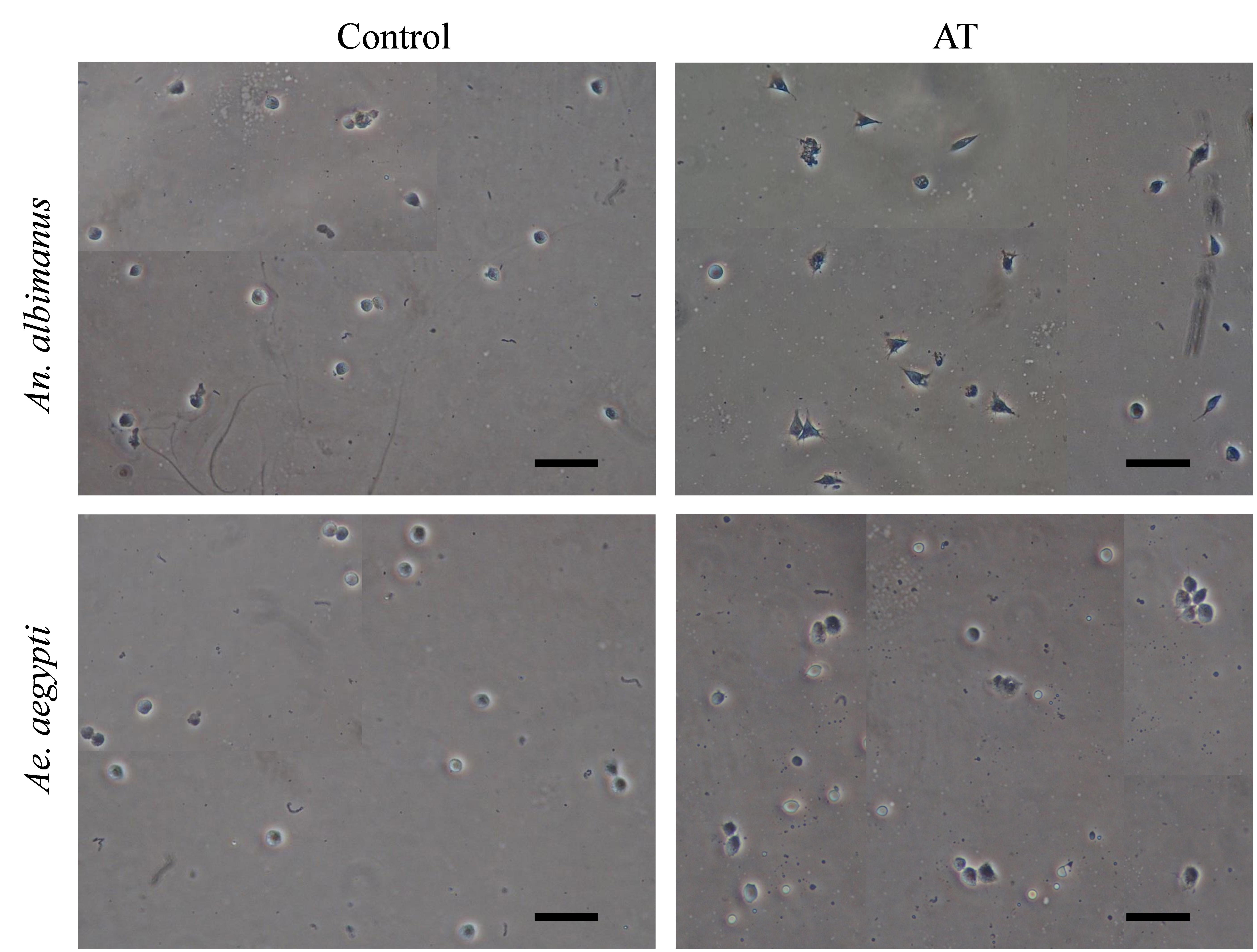

Supplement: S1 Fig — Hemocytes were obtained by perfusion and incubated in Grace’s medium alone (control) or containing AT (final concentration 10−7 M). After a 15 min incubation, samples were examined with a 20X objective by phase contrast microscopy. Morphological changes were evident in hemocytes of both mosquitoes when treated with AT. Scale bar: 40 μm. (JPG) [file pone.0175759.s001.jpg]

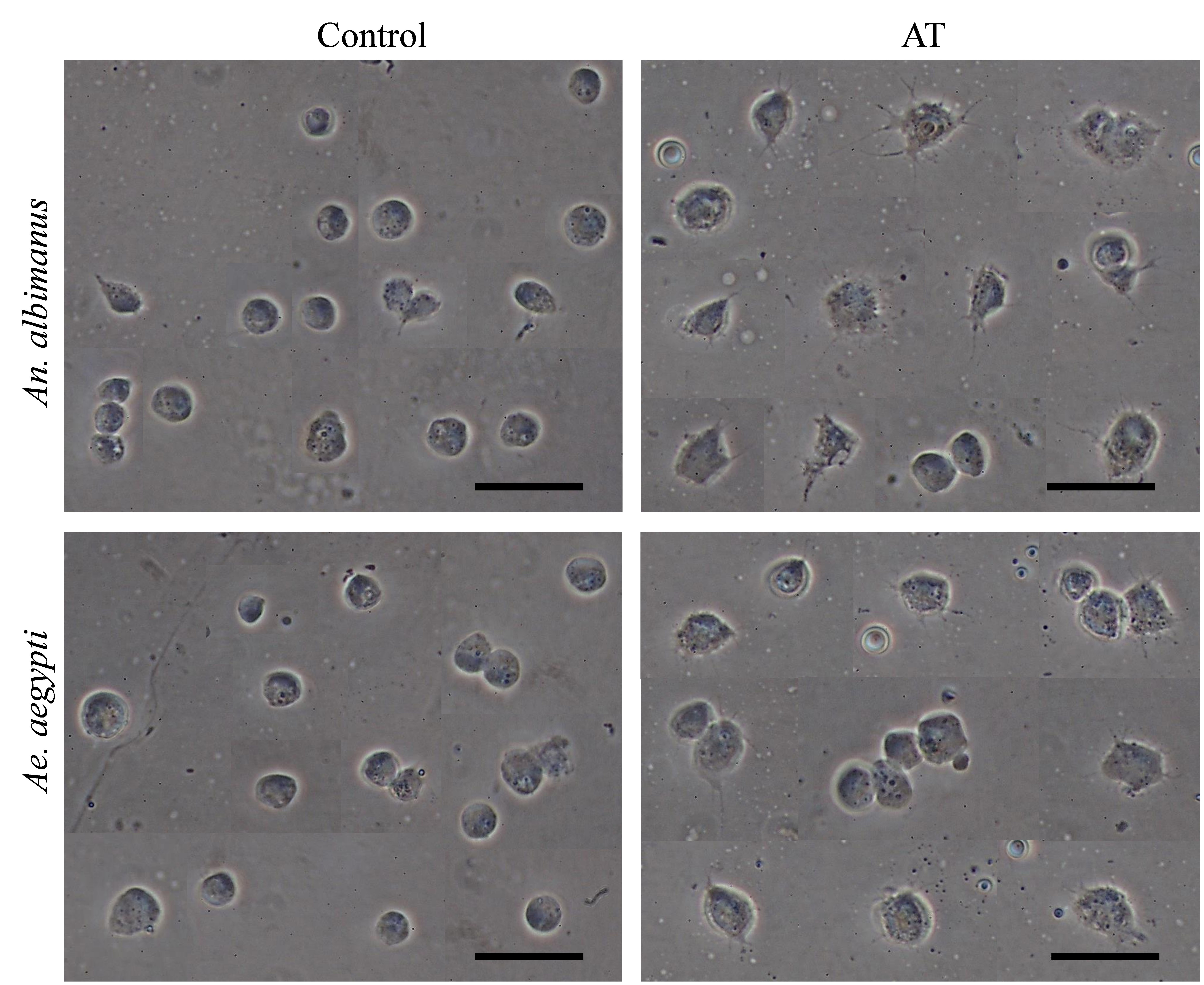

Supplement: S2 Fig — Hemocytes were obtained by perfusion and incubated in Grace’s medium alone (control) or containing AT (final concentration 10−7 M). After a 15 min incubation, samples were examined with a 100X objective by phase contrast microscopy. A threshold was arbitrary chosen; hemocytes displaying five or more filopodia were considered as “activated” (spreading). Samples were analyzed by phase contrast microscopy in a Nikon E-600 microscope (Nikon, Japan). Each panel in the figure is a composition of different fields in the samples. The presence of variable number of filopodia were evident in both mosquito hemocytes treated with AT. Scale bars: 20 μm. (JPG) [file pone.0175759.s002.jpg]

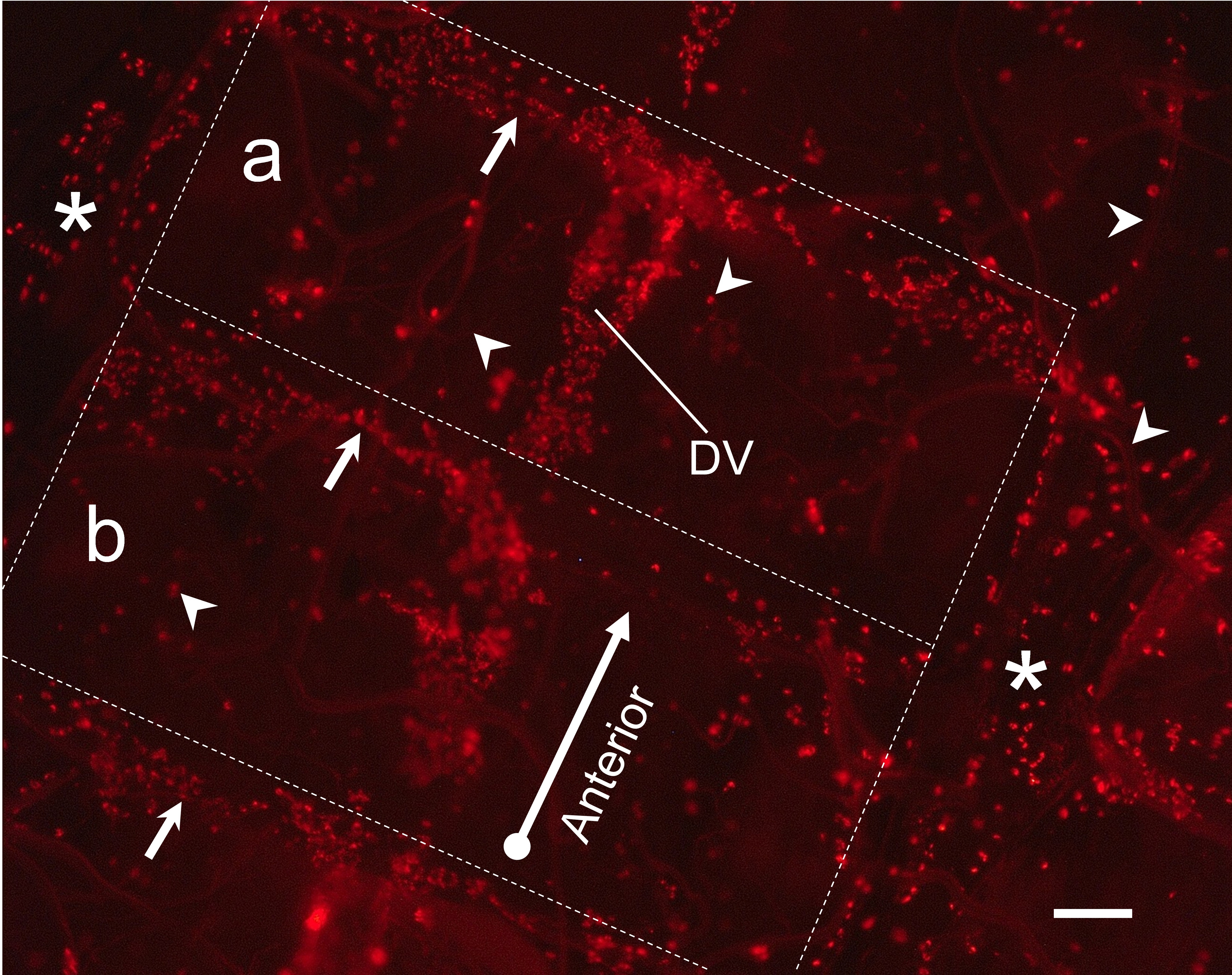

Supplement: S3 Fig — AT-QD conjugates were injected into the hemocoel and incubated for 2 h. Abdomens were dissected and analyzed by epi-fluorescence microscopy. Conjugates recognized An. albimanus hemocytes (red) distributed along the mosquito abdomen. Labeled hemocytes (arrows) were mainly accumulated on the borders of each abdominal segment (dashed boxes indicates the 2th (a) and 3th (b) abdominal segments). Hemocytes were attached to fat body, tracheoles (arrowheads) and lateral (asterisks) and inter-segmentary (arrows) pleural membranes. Dot-arrows point the abdomen anterior side. DV, dorsal vessel. Scale bar: 100 μm. (JPG) [file pone.0175759.s003.jpg]

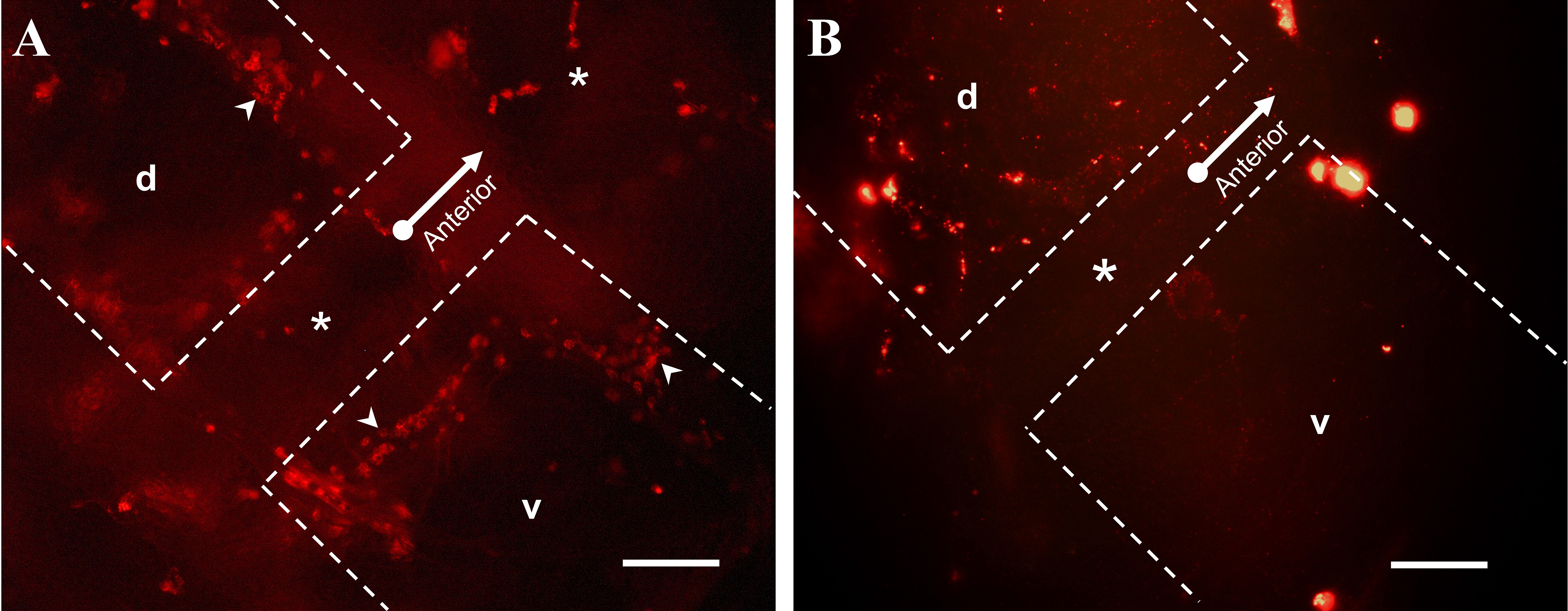

Supplement: S4 Fig — AT-QD conjugates (A) or QD-streptavidin alone (B) were injected into the hemocoel and incubated for 2 h. Abdomens were dissected and analyzed by epi-fluorescence microscopy. AT-QD conjugates recognized Ae. aegypti hemocytes (red) in the mosquito abdomen. Labeled hemocytes (arrow heads) were mainly accumulated in the borders of each abdominal segment. Hemocytes were also attached to fat body, tracheoles and lateral pleural membranes (asterisks). QD-streptavidin alone produced a diffuse background with some accumulations in non-specific regions. Dashed lines indicate section of the 5th dorsal and ventral abdominal segment. Dot-arrows point the abdomen anterior side. d, dorsal area; v, ventral area. *Lateral pleural membrane of the abdomen. Scale bars: 100 μm. (JPG) [file pone.0175759.s004.jpg]

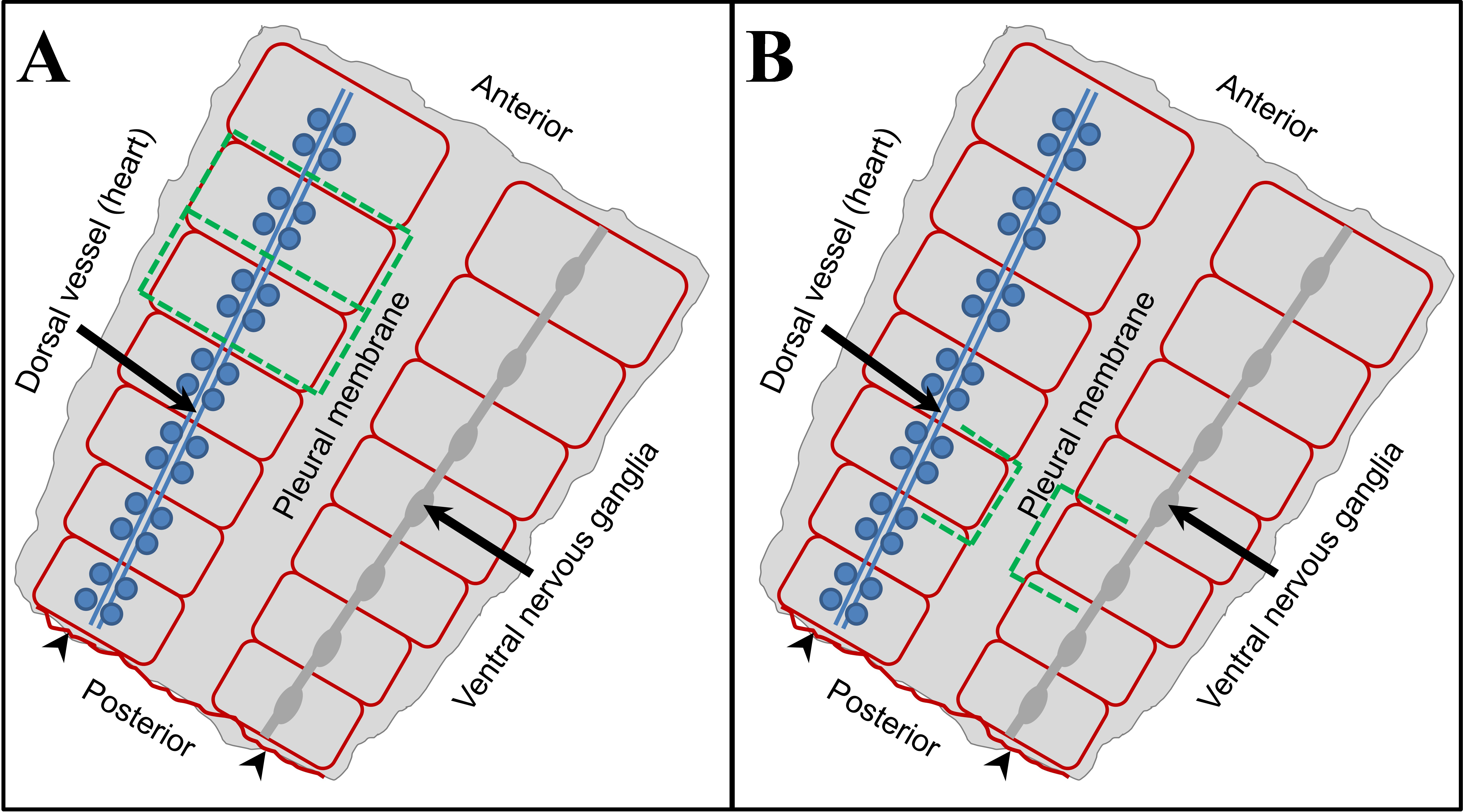

Supplement: S5 Fig — (A) Dashed box green lines correspond to the area showed in figure S3. (B) Dashed green lines correspond to the area showed in figure S4. The anterior part of the drawing represents the first abdominal segment. Arrowheads mark the area where the last (8th) abdominal segment was detached. (JPG) [file pone.0175759.s005.jpg]

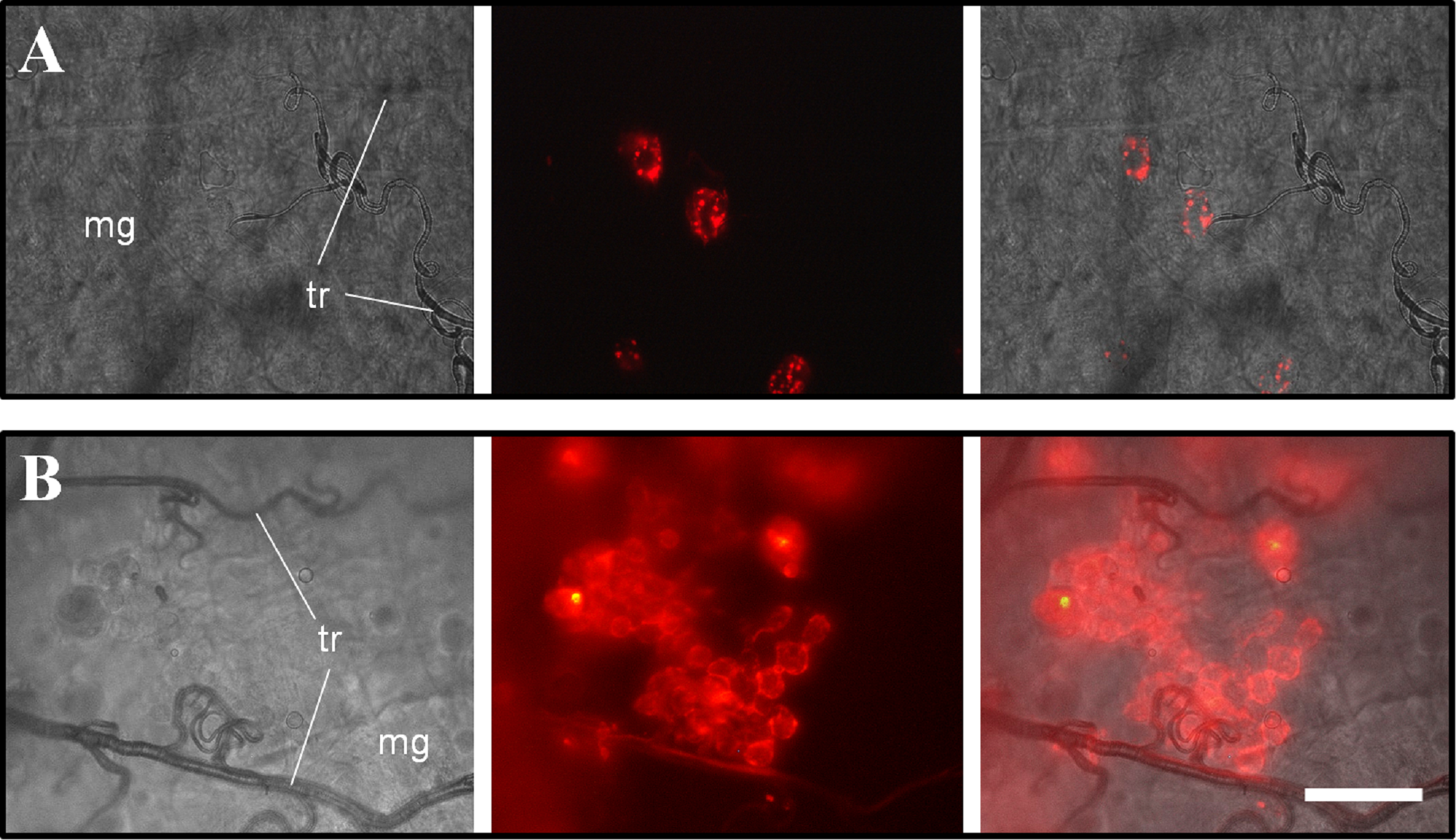

Supplement: S6 Fig — AT-QD conjugates were injected into the hemocoel and incubated for 2 h. Abdomens were dissected and analyzed by epi-fluorescence microscopy. An. albimanus (A) and Ae. aegypti (B) hemocytes attached to the midgut (mg) surface were recognized by the conjugates (red fluorescence). A and B: left, light microscopy; middle, epi-fluorescence microcopy; right, merged image. Scale bar: 20 μm. (TIF) [file pone.0175759.s006.tif]

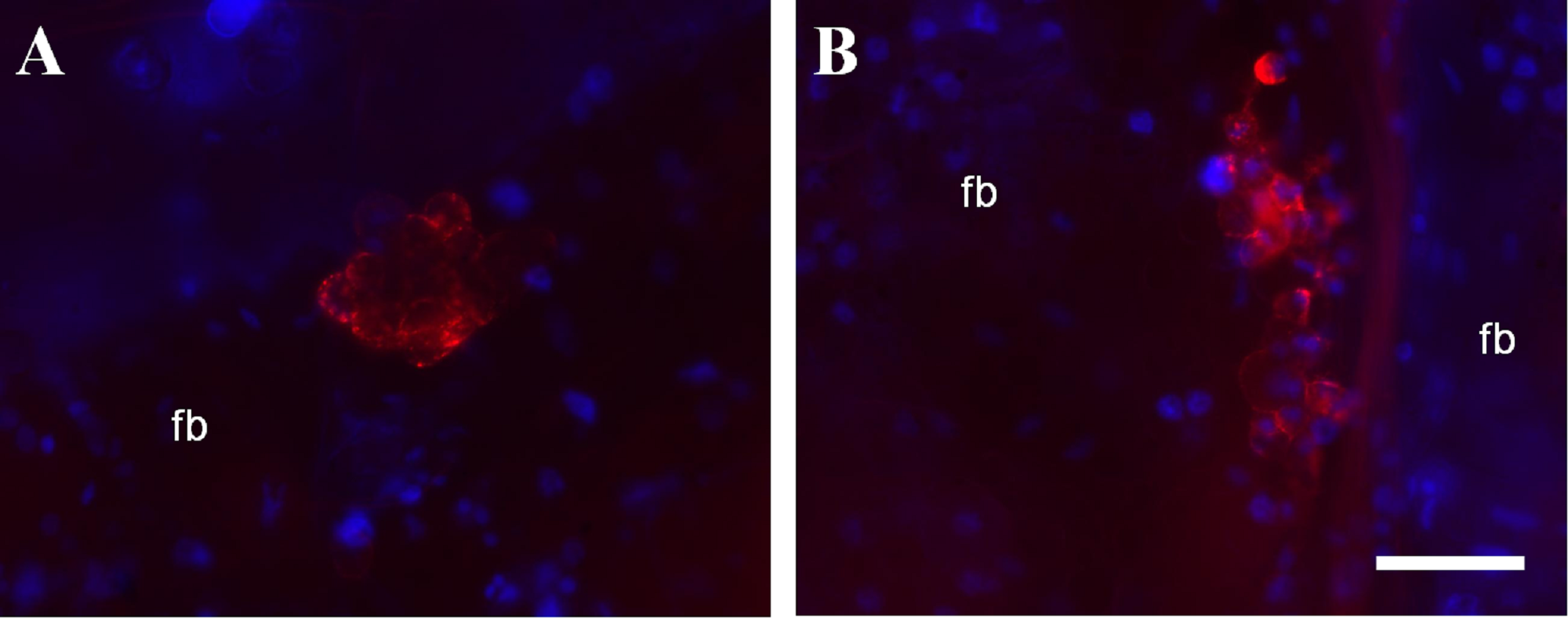

Supplement: S7 Fig — AT-QD conjugates were injected into the hemocoel and incubated for 2 h. Abdomens were dissected and analyzed by epi-fluorescence microscopy. An. albimanus (A) and Ae. aegypti (B) hemocytes are displayed attached to fat body cells (fb) (red fluorescence). Cell nuclei are stained with DAPI (blue). Scale bar: 20 μm. (TIF) [file pone.0175759.s007.tif]

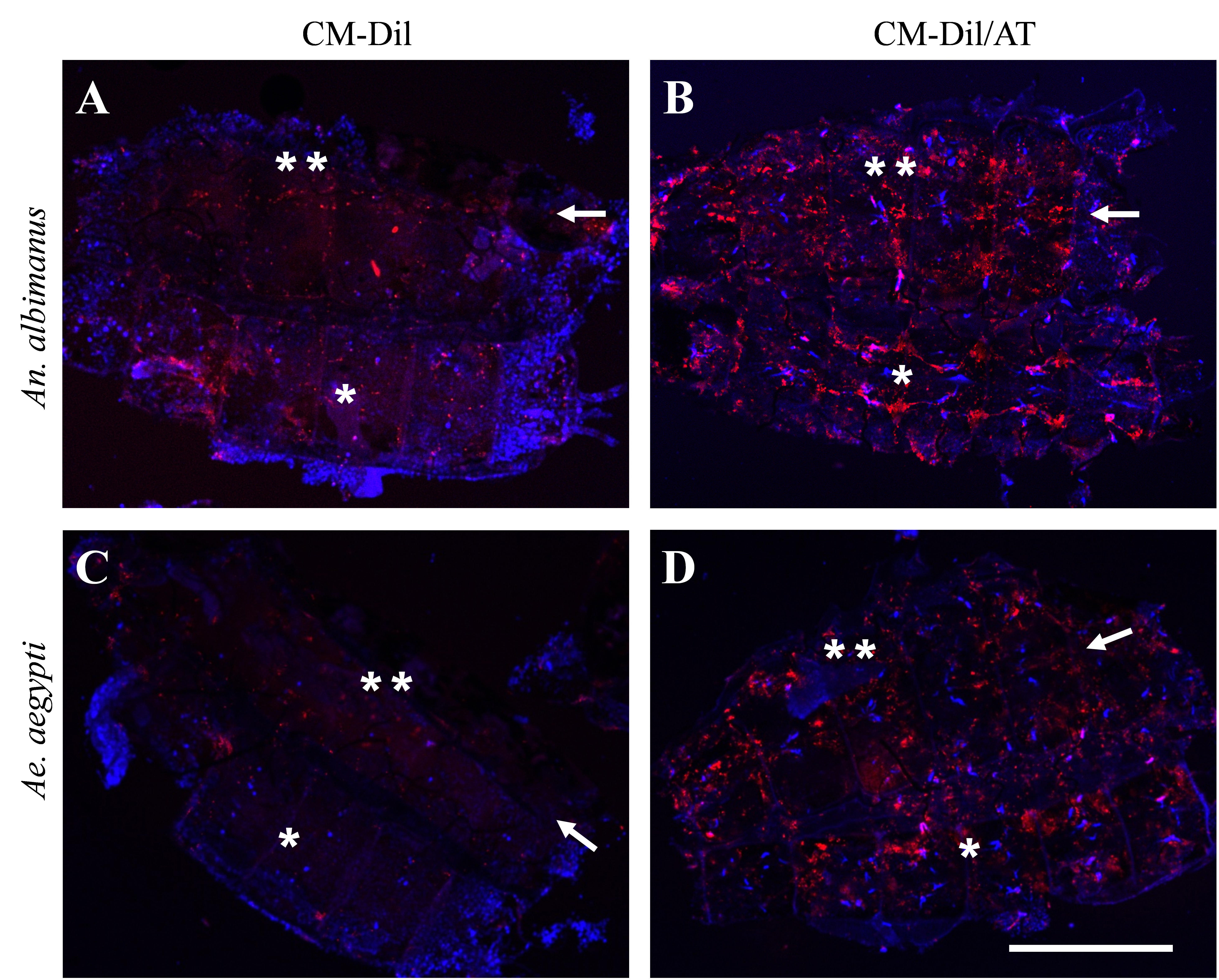

Supplement: S8 Fig — Mosquitoes were injected with CM-Dil alone (A, C) or mixed with 10−7 M of AT (B, D). After incubation, abdomens were dissected and analyzed by confocal microscopy and the images are presented as 3D views in a single projection. AT treatment increased the amount of hemocytes attached to abdominal tissues (red dots). In each panel, abdomens are orientated with the anterior side to the right. Cell nuclei are stained with DAPI (blue). Arrows: heart position. *Ventral area; **Dorsal area. Scale bar: 1 mm. (JPG) [file pone.0175759.s008.jpg]

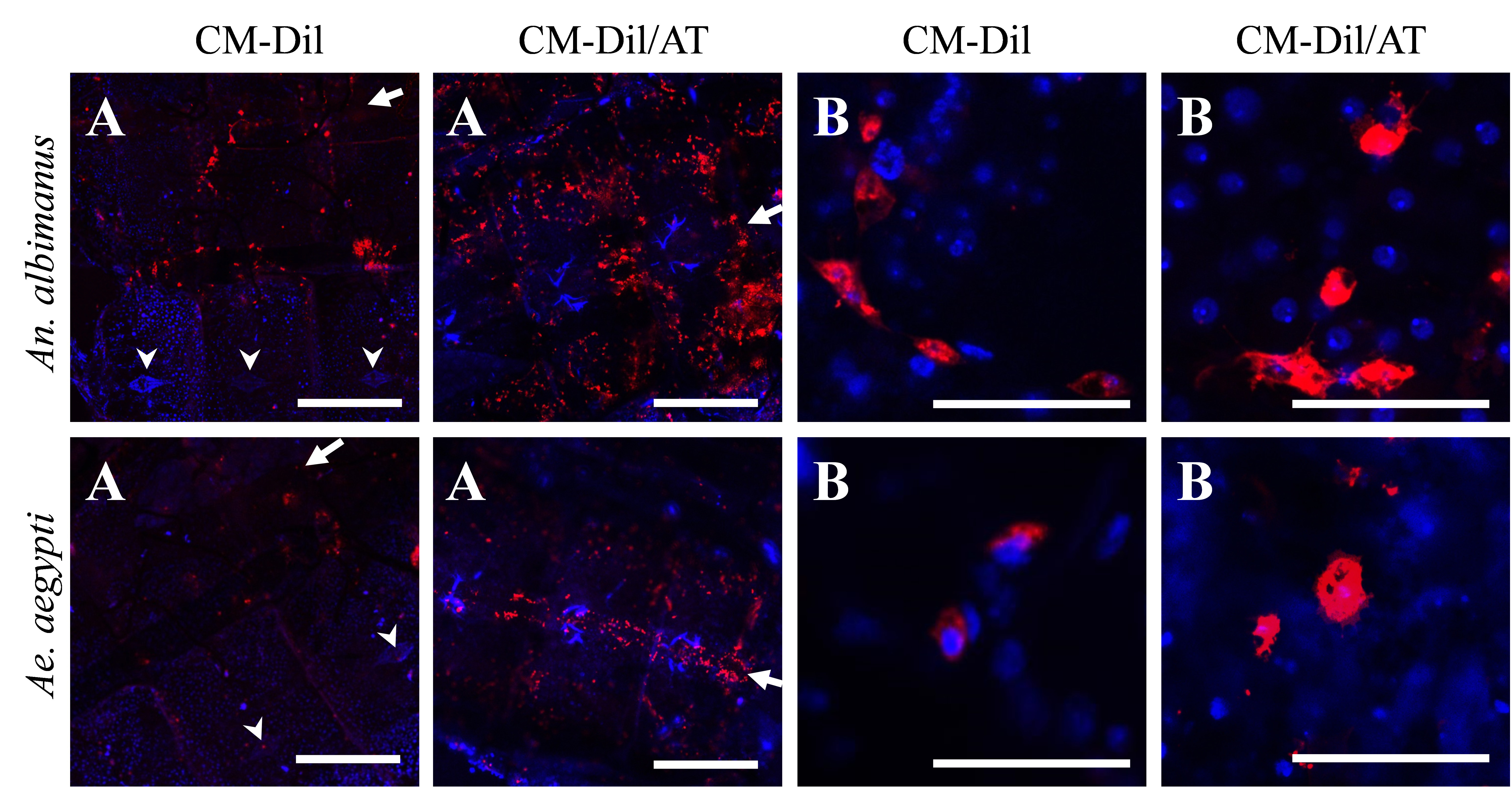

Supplement: S9 Fig — Mosquitoes were injected with CM-Dil alone or mixed with 10−7 M of AT (CM-Dil/AT). After incubation, abdomens were dissected and analyzed by confocal microscopy and the images are presented as 3D views in a single projection. AT treatment increased the amount of hemocytes (red) attached to abdominal tissues, including the heart (arrow in A). Abdomens are orientated with the anterior side to the right in panel A. (B) Detail of in vivo morphological changes in hemocytes after AT treatment. Cell nuclei are stained with DAPI (blue). Arrows: heart position. *Ventral area; **Dorsal area. Scale bar: 1 mm. (JPG) [file pone.0175759.s009.jpg]
